# The Inhibitory Receptor CLEC12A Regulates PI3K-Akt Signaling to Inhibit Neutrophil Activation and Cytokine Release

**DOI:** 10.3389/fimmu.2021.650808

**Published:** 2021-06-21

**Authors:** Guillaume Paré, Julien Vitry, Michael L. Merchant, Myriam Vaillancourt, Andréa Murru, Yunyun Shen, Sabine Elowe, Mireille H. Lahoud, Paul H. Naccache, Kenneth R. McLeish, Maria J. Fernandes

**Affiliations:** ^1^ Division of Infectious Diseases and Immunology, Laval University, Centres Hospitaliers Universitaires (CHU) de Québec Research Center, Québec, QC, Canada; ^2^ Department of Microbiology and Immunology, Faculty of Medicine, Laval University, CHU de Québec Research Center, Québec, QC, Canada; ^3^ Department of Medicine, University of Louisville School of Medicine, Louisville, KY, United States; ^4^ Department of Pediatrics, Faculty of Medicine, Laval University, CHU de Québec Research Center, Québec, QC, Canada; ^5^ Reproduction, Mother and Youth Health Division, Laval University, CHU de Québec Research Center, Québec, QC, Canada; ^6^ Department of Biochemistry and Molecular Biology, Monash Biomedicine Discovery Institute, Monash University, Clayton, VIC, Australia

**Keywords:** C-type lectin inhibitory receptor, CLEC12A protein, signaling/signaling pathways, neutrophil, inflammation, gout, rheumatoid arthritis, MAPK

## Abstract

The myeloid inhibitory C-type lectin receptor CLEC12A limits neutrophil activation, pro-inflammatory pathways and disease in mouse models of inflammatory arthritis by a molecular mechanism that remains poorly understood. We addressed how CLEC12A-mediated inhibitory signaling counteracts activating signaling by cross-linking CLEC12A in human neutrophils. CLEC12A cross-linking induced its translocation to flotillin-rich membrane domains where its ITIM was phosphorylated in a Src-dependent manner. Phosphoproteomic analysis identified candidate signaling molecules regulated by CLEC12A that include MAPKs, phosphoinositol kinases and members of the JAK-STAT pathway. Stimulating neutrophils with uric acid crystals, the etiological agent of gout, drove the hyperphosphorylation of p38 and Akt. Ultimately, one of the pathways through which CLEC12A regulates uric acid crystal-stimulated release of IL-8 by neutrophils is through a p38/PI3K-Akt signaling pathway. In summary this work defines early molecular events that underpin CLEC12A signaling in human neutrophils to modulate cytokine synthesis. Targeting this pathway could be useful therapeutically to dampen inflammation.

## Introduction

Neutrophils are critical effector cells of the innate immune response (1, 2). Collateral damage from a rapid and robust neutrophil response is mitigated by a number of counter-regulatory mechanisms, including those mediated by inhibitory receptors (3). One of the few leukocyte inhibitory receptors associated with disease is CLEC12A, a myeloid inhibitory C-type lectin receptor (CLR) (4–8). Enhanced neutrophil responses due to reduced CLEC12A plasma membrane expression following exposure to monosodium urate (MSU) crystals provided the first evidence that CLEC12A participates in the pathogenesis of an inflammatory disease (9). Similarly, the loss of CLEC12A expression in CLEC12A knock-out (KO) mice significantly increased the MSU-induced inflammatory response by enhancing leukocyte recruitment (10). CLEC12A KO mice with collagen antibody-induced arthritis also exhibited increased joint inflammation, neutrophil activation, and impaired resolution of joint injury (11). Moreover, antibody-induced CLEC12A internalization in wild-type mice enhanced collagen antibody-induced arthritis. At the molecular level, antibody-induced internalization of CLEC12A on human neutrophils enhanced the MSU-induced increase in cytoplasmic calcium and increased tyrosine phosphorylation of a number of intracellular proteins (9). Thus, similar to other inhibitory receptors, CLEC12A regulation of neutrophil responses depends on the level of plasma membrane expression (3). The data support an important regulatory role for neutrophil CLEC12A in determining disease severity in chronic inflammatory arthritis.

Significant gaps in our knowledge of the mechanisms controlling CLEC12A cell-surface expression and inhibition of neutrophil activation prevents using CLEC12A in the treatment of chronic inflammatory diseases. Inhibitory receptors typically limit cell responses by recruiting phosphatases to their immunoreceptor tyrosine-based inhibitory motif (ITIM) (12, 13). These phosphatases dephosphorylate activating signaling pathways stimulated by receptors with immunoreceptor tyrosine-based activation motifs (ITAM) (14–17). Consistent with this paradigm, antibody-induced cross-linking of CLEC12A in pervanadate-treated RAW cells induces recruitment of SHP-1 (8). The current study was designed to define signaling events associated with CLEC12A clustering and internalization in human neutrophils, and to identify the signal transduction pathways regulated by CLEC12A in neutrophils activated with stimuli relevant to inflammatory diseases. We show that MSU and antibody-mediated CLEC12A cross-linking in human neutrophils induces receptor translocation to flotillin-rich plasma membrane domains where the CLEC12A ITIM motif is phosphorylated prior to the downregulation of its expression. Decreased CLEC12A plasma membrane expression enhances MSU-induced activation of the p38-PI3K-Akt signal transduction pathway, resulting in increased neutrophil cytokine synthesis.

## Materials and Methods

### Antibodies

Two different antibodies against the HA-tag were used, namely, the anti-HA.11 (mouse monoclonal 16B12; no. 90150) from BioLegend (Pacific Heights Blvd, San Diego, CA) and the rabbit polyclonal anti-HA (NB600-363B) from Novus biologicals (Oakville, ON, Canada). The former was used to cross-link CLEC12A-HA on HEK-293T cells and the latter, for immunoblotting. A mouse IgG1 isotype antibody (no. IM0571) was obtained from Beckman Coulter (Mississauga, ON, Canada) and used as a negative control.

The monoclonal antibody clone 50C1 against an extracellular epitope of CLEC12A was generated and previously reported in Lahoud et al (18). APC-labeled anti-CLEC12A antibody (clone 50C1; no. 353606) and the mouse IgG2a isotype control (no. 401502) antibody were obtained from BioLegend (Pacific Heights Blvd, San Diego, CA). The affiniPure F(ab’)_2_ fragment goat anti-mouse IgG F(ab’)_2_ fragment specific (no. 115-006-072), horseradish peroxidase-labeled donkey anti-rabbit IgG (no. 711-035-152), horseradish peroxidase-labeled donkey anti-mouse IgG (no. 715-035-150) and Fluorescein (FITC)-AffiniPure F(ab’)_2_ fragment goat anti-mouse IgG, Fcγ fragment specific (no. 115-096-071) antibodies were obtained from Jackson ImmunoResearch Laboratories (West Grove, PA, USA). Anti-phospho-Akt (Thr308; no.9275), anti-phospho-p38 MAPK (Thr180/Tyr182; no.9216) and anti-phospho-(Ser) PKC substrate motif (no. 6967) antibodies were purchased from Cell Signaling (Danvers, MA, USA). Anti-phosphotyrosine (clone 4G10, no. 05-321) and polyclonal anti-PI3K p85 antibodies (no. 06-195) were purchased from EMD Millipore (Etobicoke, ON, Canada) and the monoclonal anti-flotillin-1 antibody (no. 610820) from BD Transduction Laboratories (Mississauga, ON, Canada). Goat anti-mouse IgG (H+L) conjugated AlexaFluor 488nm (A-11011) and the goat anti-rabbit IgG (H+L) conjugated AlexaFluor 594nm (A-10012) antibodies were purchased from Invitrogen, Thermo Fisher Scientific (Waltham, MA, USA).

The anti-phospho CLEC12A ITIM antibody was generated by injecting rabbits with a peptide composed of the amino acids within and around the ITIM motif or with a phosphorylated version of the same peptide, by Thermo Fisher Scientific (Waltham, MA, USA). Sera positive for CLEC12A reactivity were subject to a negative adsorption purification process to obtain the affinity purified anti-CLEC12A antibody that we named R-94P.

### Reagents

Sodium orthovanadate (Na_3_VO_4_), trypsin inhibitor, PMSF, OptiPrep™ density gradient medium, Nonidet P-40, Dextran T-500, aprotinin and leupeptin, methyl-β-cyclodextrin (C4555) and colchicine (C9754) were obtained from Sigma-Aldrich Canada (Oakville, ON, Canada). Percoll and protein A sepharose beads were purchased from GE Healthcare Life Science (Mississauga, ON, Canada) and Western Lightning Chemiluminescence Plus from PerkinElmer (Guelph, ON, Canada). Lymphocyte separation medium, geneticin, HEPES, fetal bovine serum (FBS) and Dulbeco’s modified Eagle’s medium (DMEM) were obtained from Wisent Bioproducts (St-Bruno, Qc, Canada) and diisopropyl fluorophosphate (DFP) from Toronto Research Chemicals (Toronto, ON, Canada). Gelatin, Tween20 and hydrogen peroxide (30%) were purchased from Fischer Scientific (Ottawa, ON, Canada) and polyethylenimine (PEI) from VWR (Mississauga, ON, Canada). Lipofectamine™ 2000 reagent, SlowFade™ Gold antifade reagent and Opti-MEM medium were purchased from Thermo Fisher Scientific (Waltham, MA, USA) and bovine serum albumin (BSA), PP2, PP3, Wortmannin and LY294002 from EMD Millipore (Etobicoke, ON, Canada). CHAPS (3-((3-Cholamidopropyl)dimethylammonio)propanesulfonic acid) was obtained from Roche Applied Science (Laval, QC, Canada) and extracellular CXCL8/IL-8 (human IL-8 cytoset, no. CHC1303) from Invitrogen (ON, Canada). Triclinic MSU crystals were synthesized and characterized as previously described by Naccache et al. (19). Latrunculin A (#10010630) was purchased from Cayman Chemical (Michigan, USA).

### Cells

Neutrophils were purified from blood donations of healthy adult volunteers collected in blood collection tubes containing sodium citrate as described in Gagné et al. (9). Isolated neutrophils were resuspended in Mg^2+^-free Hanks’ balanced salt solution (HBSS) containing 1.6 mM CaCl_2_. The entire procedure was carried out under sterile conditions. For the phosphoproteomics experiments, neutrophils were isolated using plasma-Percoll gradients followed by hypotonic lysis to remove red blood cells and resuspended in KRPB with CaCl_2_ and MgCl_2_.

HEK-293T and HeLa cells were maintained in Dulbeco’s modified Eagle’s medium (DMEM) containing 4mM L-glutamine, 1mM sodium pyruvate and 10% heat-inactivated fetal bovine serum. No antibiotics were used to culture these cell lines.

### Neutrophil Stimulation

Neutrophils (4x10^7^ cells/ml) were pre-incubated with 1mM of DFP for 10 min at room temperature prior to stimulation. For CLEC12A cross-linking, cells were incubated with 0.5µg of the 50C1 antibody or mouse isotype IgG2a/10^6^ cells for 5 min at 37°C. The cells were then washed to remove unbound antibody prior to cross-linking with 3 µg/10^6^ cells of goat anti-mouse F(ab’)_2_ anti-F(ab’)_2_ antibody for the indicated times. For MSU stimulation, neutrophils were stimulated with 3 mg/ml of MSU crystals for the indicated time at 37°C. In certain experiments, CLEC12A was cross-linked as above to induce the internalization of CLEC12A before stimulation with 3mg/ml of MSU crystals. After MSU stimulation, an aliquot of the stimulated cells was transferred at the indicated times directly into the same volume of 2X modified Laemmli’s sample buffer (composition of 1X: 62.5mM Tris-HCl (pH 6.8), 4% (w/v) SDS, 8.5% (v/v) glycerol, 2.5 mM orthovanadate, 0.025% bromophenol blue, 10μg/ml leupeptin, 10μg/ml aprotinin, 5% (v/v) β-mercaptoethanol) and heated at 95°C for 7 min. For kinetic experiments with the p38 inhibitor, cells were incubated with 10µM of SB203580 or diluent (DMSO) for 25 min at 37°C prior to stimulation with 3mg/ml MSU.

### Plasma Membrane Preparations

Neutrophils (4 × 10^7^ cells/ml) were pre-incubated with 1 mM DFP for 10 min followed by MSU stimulation or 50C1 cross-linking as described above. When indicated, cells were preincubated with 10µM of Src inhibitor PP2 or its inactive analog PP3 for 10 min at 37°C prior to the addition of MSU and terminating the stimulation in an ice bath. The cells were then subject to a quick spin (15,000 × *g*) and resuspended in cold modified relaxation buffer (100 mM KCl, 3 mM NaCl, 10 mM Hepes pH 7.4, 10 μg/ml aprotinin, 10µg/ml leupeptin, 2 mM sodium orthovanadate, 250 μg/ml trypsin inhibitor, 1 mM PMSF, 3 mM DFP) prior to sonication on ice for 22 sec at power level 1 in a Branson Sonifier 450 sonicator. Lysates were centrifuged at 400 × *g* for 2 min at 4°C and the supernatants (900 μl) added to the top of a two-step Percoll gradient composed of an equal volume (1.4 ml) of a 1.12 g/ml Percoll solution layered beneath a 1.05 g/ml Percoll solution, as described in Kjeldsen et al (20). The Percoll gradients were centrifuged for 30 min at 37,000 × *g* at 4°C in a fixed angle rotor (Beckman TLA100.4). The plasma membranes that partitioned to the upper portion of the gradient underneath the cytosol fractions were collected and diluted in modified relaxation buffer prior to a centrifugation at 100,000 × *g* for 45 min at 4°C to remove the Percoll and stored at −80°C. An aliquot was boiled for 5 min in the same volume of 2X non-reducing (without β-mercaptoethanol) modified Laemmli’s sample buffer (see above) prior to analysis.

### Isolation of Detergent-Resistant Membrane Domains

Plasma membranes freshly prepared from neutrophils (4 × 10^7^ cells/ml) incubated with isotype or CLEC12A antibody and cross-linked with an anti-F(ab’)_2_ antibody were solubilized in 1% Nonidet P-40 buffer (137 mM NaCl, 20 mM HEPES pH 7.4, 10 μg/ml aprotinin, 10µg/ml leupeptin, 2 mM sodium orthovanadate, 250 μg/ml trypsin inhibitor, 1 mM PMSF, 3 mM DPF) for 20 min on ice. Solubilized membranes were then placed on the top of a 48% OptiPrep™ cushion prepared from a stock solution (59.4% OptiPrep™, 10 mM Hepes, pH 7.4) and centrifuged at 100,000 × *g* for 1 h at 4°C in a TLA 100.4 rotor to remove soluble protein as described in Fernandes et al (21). The pellets were washed in the same buffer as above by centrifugation at 100,000 x *g* for 30 min at 4°C. Two OptiPrep™ pellets from the same donor and with the same stimulation conditions were pooled and adjusted to 40% (v/v) OptiPrep™ with a stock solution of 59.4% OptiPrep™ in 10 mM Hepes (pH 7.4). This insoluble plasma membrane preparation (700 μl) was transferred to a 4-ml centrifuge tube and overlaid with 700 μl of ice-cold solutions of 35, 30, 25, 20, and 0% (300µl) OptiPrep™ successively. The gradients were centrifuged at 38,000 × *g* for 3 h at 4°C in a TLA 100.4 rotor. Twelve fractions of 300 μl were collected from the top of the gradient and proteins were chloroform/methanol-precipitated as described previously (22). The precipitated proteins were resuspended in 35 μl of 2X non-reducing (without β-mercaptoethanol) modified Laemmli’s sample buffer (see above) and heated for 5 min at 95°C.

### Preparation of Detergent-Resistant Cell Lysate Pellets

Neutrophils were pre-incubated with 1mM of DFP for 5 min at room temperature and incubated with 2.5mM methyl-β-cyclodextrin for 30 min at 37^0^C prior to stimulation with 1mg/ml MSU for 1.5min. After a quickspin cells were resuspended in a 1% NP40 buffer, lysed for 10 min at 4^0^C and centrifuged at 13,000 × *g* for 5 min at 4°C. Cell pellets were then washed in 1X HBSS, centrifuged at 400× *g* for 2 min and the pellet resuspended in 1X sample buffer and boiled at 95°C for 10 min.

### Antibody-Induced Internalization of CLEC12A in Neutrophils

Before CLEC12A antibody-induced internalization, neutrophils (10 x 10^6^cells/ml) were incubated with the following drugs that perturb the cytoskeleton or with the diluent. Neutrophils were incubated with 10 µM colchicine (a microtubule inhibitor), 0.5µM latrunculin A (an actin filament inhibitor) or DMSO for 30 min or 5 min at 37°C, respectively. Cells were also incubated with 2.5 mM methyl-β-cyclodextrin (a cholesterol-depleting agent) for 30 min at 37°C. It is of note that at these drug concentrations, neutrophil viability is not affected, and the cells do not degranulate *(data not shown)*. Particular attention was paid to the effect of the drugs on degranulation since CLEC12A cell-surface expression was the outcome measure of these assays. Higher concentrations of methyl-β-cyclodextrin induce neutrophil degranulation. After incubation with the drugs or diluents, CLEC12A was cross-linked by incubating neutrophils with 0.1µg/10^6^ cells of 50C1 for 5min at 37°C followed by cross-linking with 0.3µg/10^6^ of a goat anti-mouse F(ab’)_2_ anti F(ab’)_2_ antibody or incubation in HBSS (negative internalization control). The extent of CLEC12A internalization was determined by flow cytometry with an anti-mouse Fc- FITC conjugated (13 µg/mL) antibody.

### Plasmid Constructs

The wild type, CLEC12A coding sequence used for our constructs corresponds to the CLEC12A isoform 2 sequence (Q5QGZ9-2 Uniprot). CLEC12A was fused to a HA tag at its C-terminus by PCR. The sequence of the forward primer used is: 5’-ATGTCTGAAGAAGTTACTTTTGCAGATC-3’, and of the reverse primer that harbors a HA-tag is: 5’-TCAAGCGTAATCTGGAACATCGTATGGGTATGCCTCCC TAAAATA TG-3’. The PCR product was ligated to the pCRII plasmid with the TA cloning kit (Thermo Fisher Scientific (Waltham, MA, USA) and then subcloned into the EcoRI site of pcDNA3 using standard molecular biology techniques. The final construct was sequenced and named CLEC12A-wt. To generate the CLEC12A-HA-Y7F construct with a mutated tyrosine in the ITIM motif (Y7F), the open reading frame of CLEC12A was amplified with the forward primer 5’-ATGTCTGAAGAAGTTACTTTTGCAGATC-3’ and the reverse primer that harbors a HA-tag 5’-TCAAGCGTAATCTGGAACATCGTATGGGTATGCCTCCC TAAAATA TG-3’. The PCR product was ligated to the pCRII plasmid and then subcloned into pcDNA3 using the same strategy as for CLEC12A-HA-wt.

### Transfection of HEK-293T and HeLa Cells

Cells were seeded at a density of 0.3 x 10^6^ cells/well (HEK 293T) or 0.45 x 10^6^ cells/well (HeLa) in 6-well plates the day prior to transient transfection with the CLEC12A-HA-wt or CLEC12A-HA-Y7F with Lipofectamine™ or PEI as per the manufacturer’s instructions. The cells were harvested 48 h post-transfection with PBS/10 mM EDTA prior to analysis. Lipofectamine™ or PEI was also used to stably transfect HEK-293T cells seeded at a density of 0.2 x 10^6^ cell/well in 6-well plates the day before transfection. Forty eight hours post-transfection, the media was changed and supplemented with 1mg/ml geneticin for 1 week and cells were sorted with an APC-labeled CLEC12A antibody (clone 50C1) by FACS (BD FACSAria II cell sorter, BD Transduction Laboratories, Mississauga, ON, Canada) and seeded in a 96-well plate at a density of 1 cell/well (**Supplementary Figure 1**). Stable cell lines were selected with 1mg/ml geneticin and thereafter the selection was maintained with 300 µg/ml geneticin.

### CLEC12A Immunoprecipitation

HEK-293T cells (1.4x10^7^ cells/ml) transiently transfected with the CLEC12A constructs were stimulated with freshly prepared pervanadate [H_2_O, orthovanadate (1mM), hydrogen peroxide (0.03%)] at a concentration of 11,1% for 10 min at 37°C in the dark. Half the cells were lysed in the same volume of 2X modified Laemmli’s sample buffer for input sample analysis. CLEC12A was immunoprecipitated from the other half of the cells as previously described in Fernandes et al (21). Briefly, cells underwent a quick spin (15,000 × *g*) and the cell pellet was resuspended in cold CHAPS buffer (10mM Tris-HCL pH 7.3, 137.2 mM NaCl, 1mM EDTA, 10µg/ml aprotinin, 10µg/ml leupeptin, 2mM sodium orthovanadate, 50µg/ml trypsin inhibitor, 1mM PMSF, 0.6% CHAPS) and lysed for 10 min on ice. Cells were then sonicated with a 3 sec pulse on ice with a Branson ultrasonic SONIFIER 450 (Branson ultrasonics corporation, Connecticut, USA) before centrifugation at 15 000 x *g* for 10 min at 4°C. The supernatants were incubated at 4°C with gentle rotation for 3 h with protein A sepharose beads previously coated with anti-CLEC12A antibody (50C1) for 1 h (2µg for immunoprecipitation of 1,4 x 10^7^ cells lysate). The beads were then centrifuged at 400 x *g* for 2 min at 4°C followed by three washing steps with cold CHAPS lysis buffer. Before the last wash, beads were divided in half, washed and resuspended in non-reducing or reducing modified Laemmli’s sample buffer 1X (see above) and incubated at 95 °C for 7 min.

### CLEC12A Cross-Linking on HEK-293T Cells

HEK-293T stably expressing CLEC12A-HA-wt or CLEC12A-HA-Y7F were harvested (10^6^ cells/100µl PBS) and incubated with the anti-HA, mouse monoclonal or the isotype antibody (3 µg/10^6^ cells) for 5 min at 37°C and centrifuged prior to cross-linking with the goat anti-mouse F(ab’)_2_ anti F(ab’)_2_ antibody (3 µg/10^6^ cells) for 10 min at 37°C. Cross-linking was stopped on ice and the cells centrifuged at 1000 x *g* for 1 min. Cell pellets were resuspended in cold NP-40 lysis buffer (10mM Tris-HCL pH 7.3, 137.2mM NaCl, 1mM EDTA, 10µg/ml aprotinin, 10µg/ml leupeptin, 2mM sodium orthovanadate, 50µg/ml trypsin inhibitor, 1mM PMSF, 1% NP-40) and incubated for 10 min on ice prior to centrifuging at 15 000 x *g* for 10 min at 4°C. An aliquot of the supernatant (SN) was incubated at 95 °C for 7 min in the same volume of non-reducing, modified 2X Laemmli’s sample buffer. The pellets were washed with cold NP-40 lysis buffer and centrifuged at 15 000 x *g* for 5 min at 4°C. Cells were sonicated with an ultrasonic pulse for 3 sec prior to the addition of non-reducing, modified 2X Laemmli’s sample buffer to the pellet and incubating at 95 °C for 7 min.

### Electrophoresis and Immunoblotting

Proteins were separated by SDS-PAGE on 10% acrylamide gels and transferred to PVDF membranes. Blocking agents and antibodies were diluted in a TBS-Tween solution (25 mM Tris-HCl, pH 7.8, 190 mM NaCl, 0.15% (v/v) Tween 20). Non-fat milk solution (5% w/v) was used to block nonspecific sites prior to immunoblotting with the anti-flotillin-1, anti-phospho-Akt, anti-CLEC12A (50C1), anti-phosphoCLEC12A (R-94P), anti-phospho-(Ser) PKC substrate and anti-HA antibodies. Gelatin solution (2%, w/v) was used to block nonspecific sites before anti-phosphotyrosine (pY), anti-phospho-p38 and anti-PI3K/p85 immunoblotting. Anti-flotillin-1 [0.125µg/ml], rabbit anti-HA [1µg/ml] and anti-CLEC12A antibody [4µg/ml] were diluted in TBS-Tween (0.15%). Anti-phosphoCLEC12A (R94-P) was diluted at 0.4-0.8µg/ml in TBS-T/BSA (5% w/v). Anti-phospho-(Ser) PKC substrate and anti-phospho-Akt were diluted 1:500 with TBS-T/BSA (5% w/v) as recommended by the manufacturer. Anti-phosphotyrosine antibody (0.25µg/ml), the anti-PI3K p85 and anti-phospho p38 (1/5000 and 1/500) were diluted in gelatin 2% as recommended by the manufacturer. Horseradish peroxidase-labeled donkey anti-rabbit IgG and horseradish peroxidase-labeled donkey anti-mouse IgG were diluted at 50ng/ml in TBS-Tween solution. Chemiluminescence reagents were used to detect antibodies within a maximal exposure time of 5 min. Equal protein loading was verified by immunoblotting against flotillin-1 or the PI3K p85 subunit.

### Confocal Microscopy

Forty-eight hours after transfecting HeLa cells with CLEC12A-HA-wt, the receptor was engaged with a rabbit anti-HA antibody (3 µg/10^6^ cells) for 7 min at 37°C followed by cross-linking with a donkey F(ab’)2 anti-rabbit F(ab’)2 antibody (4 µg/10^6^ cells) for 2 or 5 min at 37°C. For comparison, cells were also incubated with the anti-HA antibody alone. Cells were stained as previously described in Gagné et al. (23) with a few modifications. Briefly, cells were fixed in cold methanol for 20 min and washed once for 5 min at 4°C with cold PBS, and once for 5 min at room temperature. Cells were permeabilized with 0.05% Triton X-100 in PBS for 10 min at room temperature followed by an incubation for 20 min at room temperature in blocking solution (0.05% Triton X-100 in PBS supplemented with 4% FBS). Cells were then stained with the mouse anti-flotillin-1 antibody [10 µg/mL] in blocking solution overnight at 4°C prior to a wash for 30 min at 37°C and an incubation in 5µg/mL of secondary antibodies (anti-rabbit AlexaFluor 594 nm and anti-mouse AlexaFluor 488nm) for 30 min at 37°C in blocking solution. After a wash in PBS 0.05% Triton X-100 for 30 min at 37°C and several subsequent washes in water for 2 min, 90% ethanol for 1 min, and 99% ethanol for 1 min, cells were mounted with SlowFade™ Gold antifade reagent. No non-specific binding of the secondary antibodies was observed *(data not shown)*. Images were acquired at 63X with a Z-stack spacing of 0.05 µm with a CSU-X1 confocal scanner system and analyzed with the Volocity quantitation module as previously described in Gagné (23). The confocal images were deconvoluted and the point spread function that was calculated for the GFP channel and Texas Red channel was applied using velocity module for iterative restoration. The co-localization module was used to determine the degree of co-localization between CLEC12A and flotillin-1 by calculating the Pearson’s linear correlation coefficient.

### Phosphoproteomics and Analysis

Neutrophils were suspended in KRPB containing calcium and magnesium and incubated with or without TNF-α (2 ng/ml) and with or without 50C1 for 5 or 10 min at 37°C prior to cross-linking with a goat F(ab’)2 anti-mouse F(ab’)2. Following stimulation, cells were pelleted immediately at 4000 rpm for 1 min at 4°C and lysed by resuspending the pellet in ice-cold extraction buffer (20 mM Tris-HCl pH 7.8, 10 mM HEPES, 25 mM NaCl, 2 mM EDTA, 10 mM EGTA), and 1% (w/w) protease inhibitor cocktail, followed by sonication using 3 to 5 cycles at room temperature. Cell debris and nuclei were removed by centrifugation at 700 x *g* for 10 min at 4°C. The supernatant was transferred to ultracentrifugation tubes and centrifuged at 100,000 x *g* for 30 min at 4°C. After centrifugation, the supernatant was stored at -80°C until used. The phosphoproteomics was performed as previously described in McLeish et al. (24).

Samples were reduced, alkylated, and trypsinized and phosphopeptides enriched using sequential TiO2 and immobilized metal affinity chromatography chromatographic steps to purify polyphosphorylated peptides and monophosphorylated peptides, as described previously (24). The effects of co-isolation of non-phosphorylated peptides enriched in aspartic acid and/or glutamic acid residues were minimized with the use of 1 M glycolic acid as a competitive ligand for the TiO2 step. Targeted analysis of phosphopeptide fractions was achieved using a nanoflow ultra HPLC/nanospray- Linear Ion Trap-Orbitrap Elite mass spectrometer with collision-induced dissociation and electron transfer dissociation fragmentation in a bottom-up approach.

Acquired data were analyzed against human Reference Sequence (Human-Ref131014.fasta) and decoy databases using Sequest HT by PD1.4, considering tryptic cleavage, maximum of 2 missed cleavages per peptide, and a mass error of 50 ppm in precursor ions of a specific mass-to-charge ratio and 1.2 Da in fragmented precursor ions data. The searches considered a maximum of 4 modifications to any peptide and the modifications of cysteine (carbamidomethyl/+57.021 Da), methionine (oxidation/+15.995 Da), and serine/threonine/tyrosine (phospho/+79.966 Da). PD1.4 data were filtered first to retrieve all peptides containing serine-, threonine-, or tyrosine containing peptides (putative kinase-targetable peptides). Peptide grouping was enabled by mass and sequence. Protein grouping was enabled to consider only PSMs with confidence at least low/medium/high-confidence peptides and to consider proteins only with PSMs having a delta correlation better than 1.0. These data were filtered to eliminate entries where a low-confidence assignment was the only assignment present in any condition. If medium- or high-confidence peptides were present in 1 condition, then the peptide areas were exported for all conditions. For this purpose, medium-confidence peptides had minimal xcorr values for charge +2. 0.9, charge +3. 1.2, and charges +4–7. 1.5, and for high-confidence peptides, the corresponding xcorr values were for charge +2. 2, charge +3. 2.5, and charges +4–7.3. These peptides were exported to an Excel spreadsheet for peptide grouping by mass and sequence and manual comparison of abundance between treatment and control conditions.

### Enzyme-Linked Immunosorbent Assays

Extracellular CXCL8/IL-8 was quantified by ELISA. Briefly, neutrophils (2x10^7^ cells/ml) in RPMI without phenol red were incubated with the indicated concentrations of compounds or diluent (DMSO) for 10 min at 37°C prior to the addition of buffer or 1 mg/ml MSU. Neutrophil-MSU contact was synchronized by centrifuging at 400 x *g* for 10 seconds. After a 3 h incubation at 37°C, cells were centrifuged at 400 × *g* for 2 min and the supernatants harvested and clarified with a centrifugation at 16,000 x g for 5 min at 4°C. Each condition was measured in duplicate (Spectramax 190 plate reader) prior to quantifying IL-8 as per the manufacturer's instructions.

### Statistical Analysis

Numerical values are means ± SEM. Statistical analyses were either performed using one-way ANOVA followed by Fisher or Dunn’s multiple comparisons tests, or two-way ANOVA followed by the Turkey’s multiple comparisons test. Calculations were made with GraphPad Prism 6 software (GraphPad Software, La Jolla, CA, USA). Significance was considered at a value of P < 0.05.

## Results

### CLEC12A Cross-Linking Induces Its Translocation to Detergent-Resistant Membrane Domains

**Figure 1:**
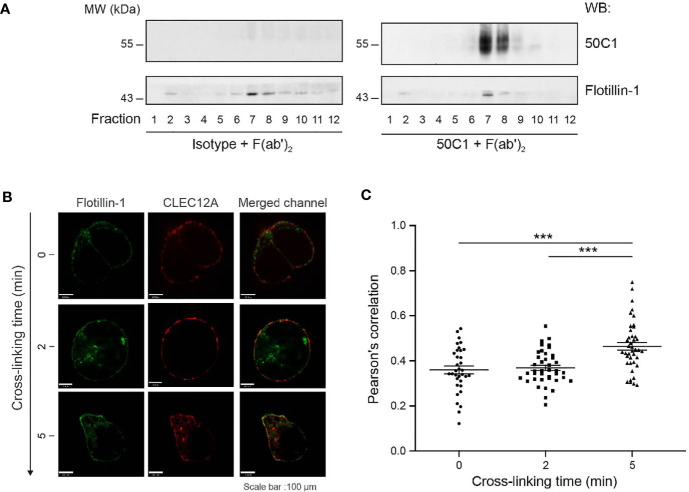
CLEC12A translocates to detergent-resistant membrane domains after antibody-induced cross-linking. **(A)** Detergent-resistant membrane domains were isolated from plasma membrane preparations from human neutrophils incubated with an isotype antibody or 50C1 prior to cross-linking. Fractions were migrated on a non-reducing acrylamide gel and immunoblotted with 50C1 or flotillin-1. Data are representative of 3 independent experiments. **(B)** Co-localization of CLEC12A and flotillin-1 was determined in HeLa cells transiently expressing CLEC12A-HA-wt after cross-linking with an anti-HA antibody or incubation with the anti-HA antibody alone. Data are representative of 3 independent experiments. **(C)** The extent of co-localization was determined by Pearson R values (mean ± SEM) for the different cross-linking times. Statistical analysis: one way ANOVA and Dunn’s test were performed to compare cross-linked condition to control cells. ***P < 0.001.

To understand the molecular events initiating CLEC12A internalization and regulation of neutrophil activation, we determined receptor distribution in plasma membrane domains known to facilitate cellular responses (25). These membrane domains are detergent-resistant and enriched for signaling and structural proteins as well as flotillin. Detergent-resistant domains were isolated from a preparation of neutrophil plasma membranes devoid of soluble protein, by differential centrifugation. Localization of CLEC12A in flotillin-rich membrane domains was determined by Western blotting of detergent-resistant, plasma membrane fractions obtained from neutrophils with or without receptor cross-linking with the CLEC12A-specific antibody, 50C1, followed with a secondary antibody (9). Flotillin-1 was primarily located in fractions 7 and 8 (**Figure 1A**). Cross-linking resulted in marked enrichment of CLEC12A in neutrophil plasma membrane fractions containing flotillin-1 compared to membrane fractions of neutrophils after cross-linking with isotype antibody that are devoid of the receptor.

Translocation of cross-linked CLEC12A to flotillin-1-containing membrane domains was confirmed by confocal microscopy of HeLa cells. HeLa cells transiently transfected with HA-tagged CLEC12A (CLEC12A-HA-wt) express CLEC12A on their surface and internalize the receptor following cross-linking (**Supplementary Figure 1** *and data not shown*). CLEC12A-HA-wt co-localized with flotillin-1 in HeLa cells after cross-linking with an anti-HA and secondary antibody, but not in cells exposed to anti-HA antibody alone (**Figure 1B**). The observed co-localization between CLEC12A-HA-wt and flotillin-1 is significant as determined by the R Pearson’s correlation coefficient after 2 min (**Pearson R=0.4524 ± 0.01769 (SEM)) and 5 min (**Pearson R=0. 5214 ± 0. 04337 (SEM)) of cross-linking (***P < 0.001) (**Figure 1C**).

**Figure 2:**
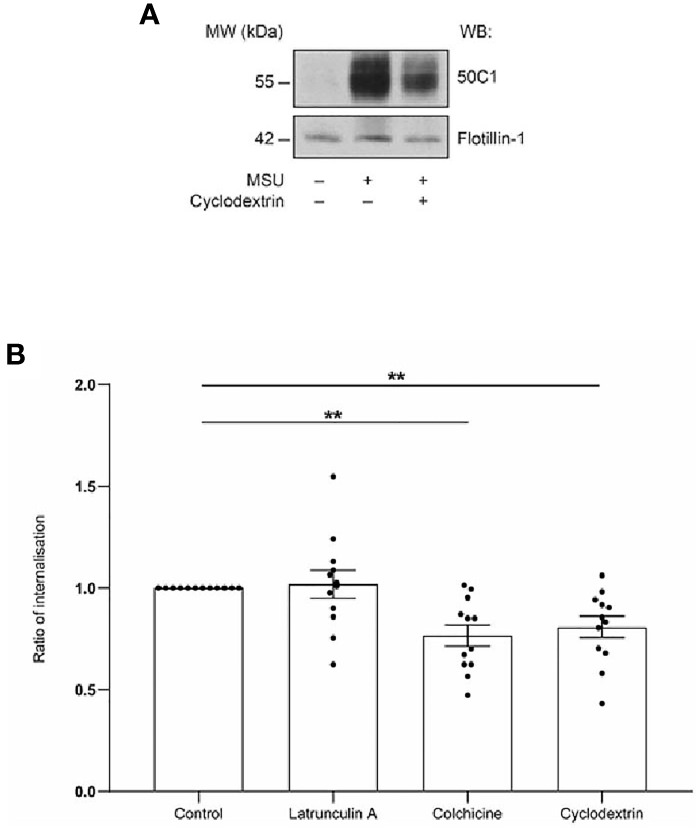
Antibody-induced cross-linking and internalization of CLEC12A is dependent on cholesterol-rich, membrane domains and microtubules. **(A)** Methyl-β-cyclodextrin-treated neutrophils were lysed in cold, 1% Nonidet P-40 after MSU stimulation and the insoluble pellet of the lysate immunoblotted with 50C1. These data are representative of 3 independent experiments. **(B)** Cell-surface CLEC12A expression of neutrophils treated with all compounds or diluent (*control*) was determined by flow cytometry after CLEC12A cross-linking. These data are representative of 12 independent experiments (Mean± SEM). Statistical analysis: One way ANOVA and Fischer’s LSD. ** P < 0.01.

The data in **Figure 1** demonstrate that CLEC12A translocates prior to internalization to flotillin-1-containing membrane domains that are known to be enriched in cholesterol. To determine if translocation to these membrane domains is necessary for receptor internalization, the effect of cholesterol-depletion on CLEC12A translocation and internalization was determined after MSU stimulation or receptor, antibody-induced cross-linking in human neutrophils. We previously reported that CLEC12A is internalized in MSU-stimulated neutrophils (9). Methyl-β-cyclodextrin induced a significant reduction in CLEC12A translocation to flotillin-enriched, detergent-resistant cell pellets in neutrophils stimulated with MSU (**Figure 2A**). Translocation to flotillin-enriched, detergent-resistant cell pellets was analyzed as MSU perturb the gradient used to isolate detergent-resistant membrane domains. Consistent with this observation, methyl-β-cyclodextrin also significantly inhibited the antibody-induced internalization of CLEC12A (**Figure 2B**). CLEC12A displacement in plasma membranes is thus induced by clustering and its internalization dependent on the integrity of cholesterol-rich, flotillin membrane domains.

Flotillins associate with the cytoskeleton and play a key role in endocytosis (26). The role of microtubules and the actin cytoskeleton on CLEC12A internalization was thus investigated by incubating neutrophils with colchicine and latrunculin A prior to receptor cross-linking. Colchicine significantly inhibited CLEC12A internalization, while latrunculin A had no effect (**Figure 2B**). Microtubules, but not the actin cytoskeleton, are thus required for CLEC12A internalization.

### CLEC12A Is Phosphorylated Following Translocation

**Figure 3:**
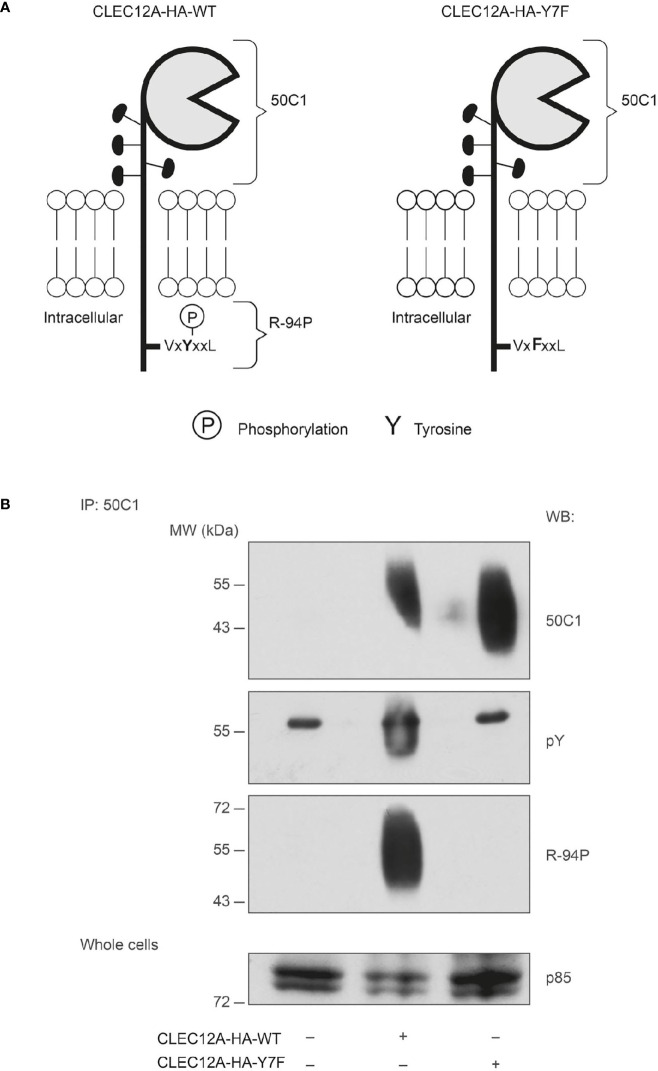
Antibody-induced cross-linking of CLEC12A induces the phosphorylation of its ITIM in HEK-293T cells. **(A)** A schematic diagram of the wild-type (CLEC12A-HA-wt) and mutant (CLEC12A-HA-Y7F) constructs. Regions of the receptor recognized by 50C1 and the anti-CLEC12A ITIM phopsho-antibody, R-94P, are indicated by brackets. **(B)** HEK-293T cells transiently transfected with CLEC12A-HA-wt or CLEC12A- HA-Y7F were treated with pervanadate prior to immunoprecipitating with 50C1 and immunoblotting with 50C1, R-94P, an anti-phospho-tyrosine (*pY*) antibody or an anti-p85 subunit of PI3K (*p85*) antibody (*loading control*). Data are representative of 3 independent experiments.

To follow CLEC12A ITIM phosphorylation, an antibody that recognizes the phosphorylated ITIM of CLEC12A was developed (**Figure 3A**). The affinity purified antibody (R-94P) bound a phosphorylated version of a peptide composed of the ITIM of CLEC12A, but not the non-phosphorylated form of the peptide, as determined by dot blot *(data not shown)*. To confirm the specificity of R-94P for the phosphorylated form of the CLEC12A ITIM, HEK-293T cells were transfected with CLEC12A-HA-wt or CLEC12A in which the tyrosine of its ITIM is substituted with a phenylalanine (CLEC12A-HA-Y7F). The phosphorylation of CLEC12A-HA-wt immunoprecipitated from pervanadate-treated HEK-293T cells was detected with an antibody against phosphorylated tyrosine residues (pY) and with R-94P (**Figure 3B**). In contrast, neither antibody recognized CLEC12A-HA-Y7F. R-94P thus recognizes the phosphorylated ITIM of CLEC12A.

**Figure 4:**
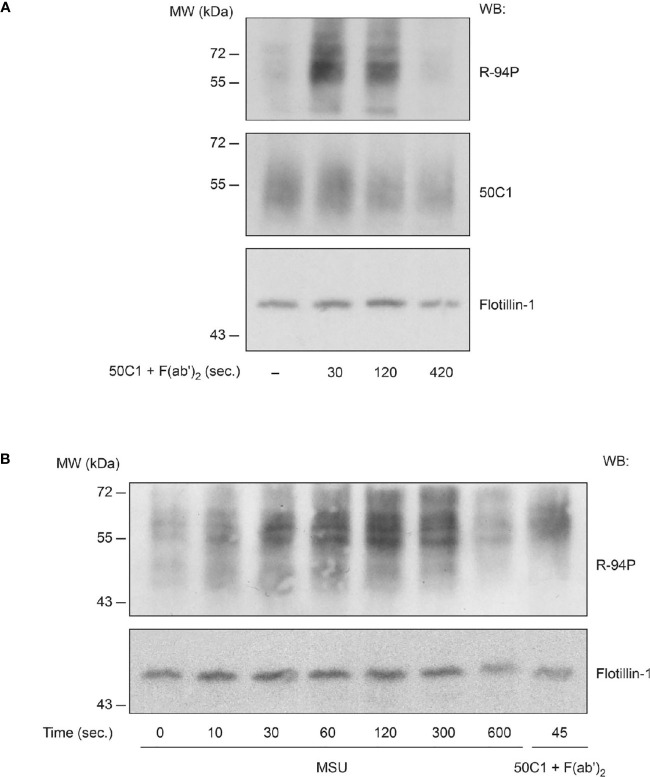
CLEC12A phosphorylation upon antibody-induced cross-linking and MSU stimulation in human neutrophils. **(A)** Cell-surface CLEC12A was cross-linked with 50C1 on human neutrophils prior to immunoblotting plasma membrane preparations with R-94P, 50C1 or flotillin-1 (*loading control*) antibodies. Data are representative of 3 independent experiments. **(B)** Plasma membranes isolated from MSU-stimulated neutrophils were immunoblotted with R94P or flotillin-1. For comparison, CLEC12A was also cross-linked as in **(A)** for 45 seconds. Data are representative of 3 independent experiments.

As inhibitory receptors signal through the ITIM cytoplasmic motif (12), the phosphorylation of the CLEC12A ITIM was examined in human neutrophils. CLEC12A was cross-linked with 50C1 prior to Western blot analysis with R-94P of plasma membrane preparations. Minimal CLEC12A phosphorylation was detected in the plasma membrane of resting human neutrophils (**Figure 4A** and **Supplementary Figure 2**). In contrast, CLEC12A phosphorylation was markedly enhanced at 30 seconds and 120 seconds after cross-linking, and returned to basal levels by 7 min. Since MSU-stimulated neutrophils internalize CLEC12A, the ability of MSU to stimulate the phosphorylation of the CLEC12A ITIM was examined in human neutrophils. **Figure 4B** shows a time course of MSU-induced CLEC12A phosphorylation in neutrophil plasma membrane preparations. R-94P immunoblotting detected phosphorylation above basal levels within 10 seconds of MSU stimulation that peaked between 1 and 2 min, and returned to basal levels by 10 min. A time-dependent phosphorylation of CLEC12A’s ITIM is thus induced by MSU and 50C1 cross-linking.

**Figure 5:**
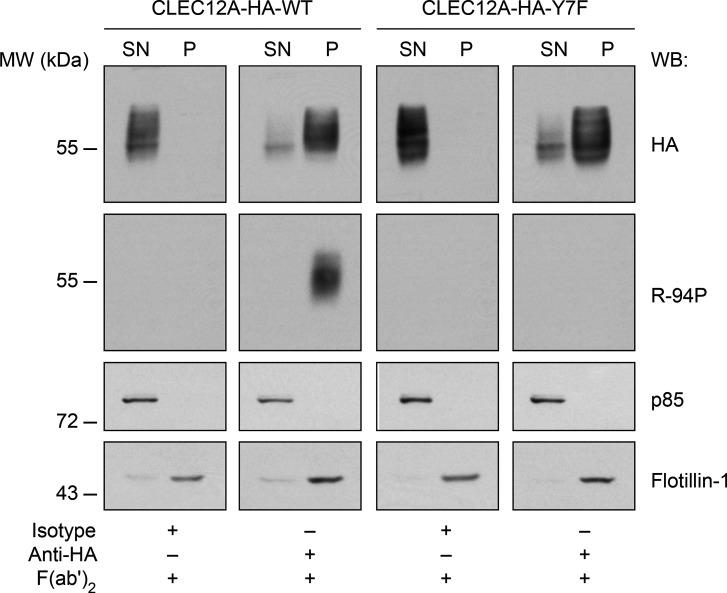
CLEC12A is phosphorylated upon its antibody-induced translocation to the flotillin-rich, detergent-insoluble cell pellet. Proteins in the flotillin-enriched pellet (*P*) and supernatant (*SN*) of cell lysates of HEK-293T cells stably transfected with CLEC12A-HA-wt or CLEC12A-HA-Y7F after cross-linking with an anti-HA antibody were immunoblotted with an anti-HA, R-94P, anti-PI3K p85 subunit (*p85*) or flotillin-1 antibodies. The p85 protein was a loading control for proteins in the (*SN*) and flotillin-1 for proteins in (*P*). Data are representative of 3 independent experiments.

The relationship of ITIM phosphorylation to translocation of CLEC12A into flotillin-1-containing membrane domains was then examined by cross-linking CLEC12A-HA-wt or CLEC12A-HA-Y7F with anti-HA and secondary antibodies in transiently transfected HEK-293T cells as neutrophils are not amenable to transfection. In cells incubated with isotype antibody, CLEC12A-HA-wt was only detected in the soluble fractions of cell lysates. CLEC12A-HA-wt cross-linking resulted in translocation to the flotillin-1 enriched, detergent-resistant cell pellet (**Figure 5**, *two left panels*). Western blot with R-94P showed ITIM phosphorylation of CLEC12A-HA-wt only within flotillin-1 enriched, cell pellets following receptor cross-linking. Cross-linking CLEC12A-HA-Y7F also resulted in translocation from the soluble to the flotillin-1 enriched cell pellet. Phosphorylation of the CLEC12A ITIM thus occurs after receptor cross-linking and translocation to flotillin-rich membrane fractions. Moreover, CLEC12A phosphorylation is not required for CLEC12A translocation to flotillin-rich, membrane fractions.

**Figure 6:**
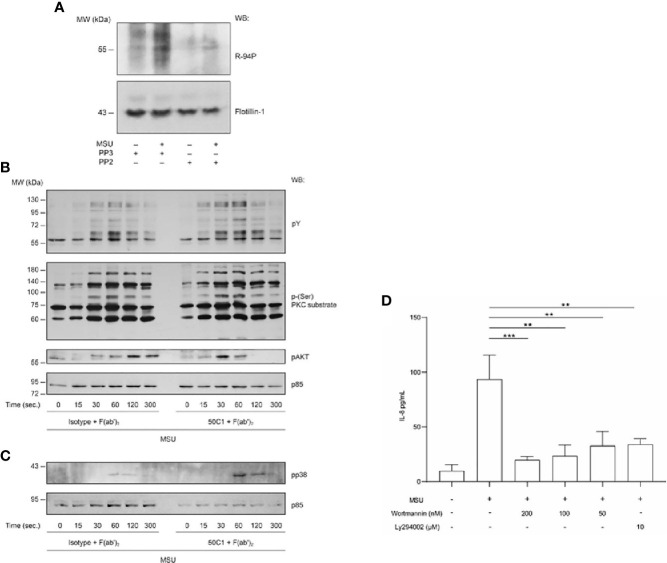
Antibody-induced cross-linking of CLEC12A enhances the phosphorylation of tyrosine residues, PKC substrates, Akt and p38 to regulate MSU-induced IL-8 production in human neutrophils. **(A)** Plasma membranes were isolated from neutrophils incubated with PP2 or its inactive analog PP3 prior to MSU stimulation and immunoblotting with R-94P or an anti-flotillin-1 antibody (*loading control*). Data are representative of 3 independent experiments. **(B, C)** CLEC12A was cross-linked on neutrophils with 50C1 prior to MSU stimulation and cell lysates immunoblotted with the indicated antibodies and the loading control antibody (anti-PI3K p85 subunit). pY = anti-phosphotyrosine antibody. Data are representative of 3 independent experiments. **(D)** CXCL8/IL-8 released by MSU-stimulated neutrophils in the presence or absence of Wortmannin, Ly294002 or dilutant (DMSO) was determined by ELISA. Data are representative of 3 independent experiments. Statistical analysis: Two-way ANOVA and the Turkey’s multiple comparisons test was performed to compare the treated cells to the control cells. **P < 0.01; ***P < 0.001.

As Src kinases are known to phosphorylate ITIM motifs (14), the ability of the Src kinase inhibitor PP2 to prevent CLEC12A phosphorylation in human neutrophils was examined. Western blot analysis with R-94P of plasma membranes prepared from neutrophils stimulated with MSU shows that PP2, but not the inactive analog PP3, prevented phosphorylation of CLEC12A (**Figure 6A**). Taken together, our data indicate that following clustering, CLEC12A translocates to flotillin-rich plasma membrane domains of neutrophils where the ITIM region is phosphorylated in a Src-dependent manner.

### Reduced Expression of Cell-Surface CLEC12A Enhances MSU-Stimulated Kinase Activity in Human Neutrophils

We previously reported several signaling events induced by MSU in neutrophils, one of which is regulated by CLEC12A, the tyrosine phosphorylation of intracellular substrates (9, 27). To identify additional signaling events regulated by CLEC12A in activated neutrophils, the effect of reduced CLEC12A expression on MSU-induced protein phosphorylation was examined. Western blotting of lysates prepared from neutrophils stimulated with MSU after antibody-induced cross-linking of CLEC12A showed enhanced MSU-induced tyrosine phosphorylation of proteins larger than 72kD compared to neutrophils incubated with control isotype antibody confirming our previous observation (**Figure 6B**, *pY blot*). A similar observation was made for serine phosphorylation of PKC substrates as early as 15 sec (**Figure 6B**, *p-(Ser)-PKC substrate blot*). We interpret these results to indicate that cell-surface CLEC12A expression required for counter-regulatory function in MSU-activated human neutrophils is associated with inhibition of both tyrosine kinase and PKC activity.

### Proteomic Analysis of Protein Phosphorylation After CLEC12A Cross-Linking on Human Neutrophils

Based on the regulation of protein phosphorylation by CLEC12A, an unbiased phosphoproteomic analysis was performed to identify candidate phosphorylation events induced by CLEC12A cross-linking in neutrophils. A total of 9367 phosphopeptides were identified from two separate experiments representing 3089 unique proteins. Proteins phosphorylated in response to CLEC12A cross-linking were identified by subtraction analysis of phosphopeptides detected from cells incubated with isotype antibody from those identified after 50C1 cross-linking. A total of 2033 phosphopeptides, representing 1259 unique proteins, were identified from neutrophils after CLEC12A cross-linking, but were absent in cells incubated with isotype antibody incubation. An additional 183 phosphopeptides from 120 unique proteins were present at a 2-fold higher abundance after cross-linking, compared to isotype control. Gene Ontology enrichment analysis for molecular function was performed on those 1379 proteins with enhanced phosphorylation following CLEC12A crosslinking. **Supplementary Table 1** lists the 9 molecular functions of those phosphoproteins with statistically significant enrichment, including proteins with MAP kinase and GTPase activity and proteins involved in phospholipid binding, adhesion, and actin binding. STRING analysis of proteins with differential phosphorylation predicted 62 proteins that possessed kinase or phosphatase activity (**Supplementary Table 2**). Analysis of interactions among those 62 proteins by STRING defined three clusters of signaling proteins that might define relevant signal transduction pathways activated by CLEC12A cross-linking (**Supplementary Figure 3**). The cluster with the largest number of proteins contained components of the MAPK2 (ERK1) and MAPK8 (JNK1) signaling cascades and protein kinase C beta and delta. Another cluster contained ephrin receptors and ligands, and the tyrosine kinase BLK. The third cluster contained the catalytic subunit of PI3K and two subunits of AMPK.

As CLEC12A regulates phosphorylation of kinase substrates (**Figure 6**), we also screened for signal transduction pathways in stimulated human neutrophils after the antibody-induced down-regulation of CLEC12A. Based on the importance of TNF-α in rheumatoid arthritis (28) and gout (29), subtraction analysis of phosphoproteins generated by stimulation with TNF-α after cross-linking CLEC12A from those generated by TNF-α alone was performed. A total of 1884 phosphopeptides representing 1400 unique proteins were absent or showed a 2-fold or greater reduction in TNF-α stimulated cells in which CLEC12A internalization was induced by cross-linking. Gene Ontology enrichment analysis for molecular function of those 1400 proteins is shown in **Supplementary Table 3**. The categories of proteins showing reduced phosphorylation with CLEC12A internalization included cytoskeletal structural and regulatory proteins and protein kinases with both tyrosine kinase and serine/threonine kinase activity. STRING analysis of all 1400 proteins identified 94 proteins with kinase or phosphatase activity (**Supplementary Table 4**). STRING cluster analysis identified two major clusters (**Supplementary Figure 4**). The first, centered on MAPK8 (JNK1) contained the tyrosine kinases LYN, JAK3, and ABL1 and the serine/threonine kinases PAK1, PKC zeta, and ATM. The second cluster centered on the tyrosine kinase FYN contained tyrosine kinases JAK2, CSK and Ephrin receptors, PAK1, the catalytic subunit of PI3K, and the Akt activating kinase PKD1.

### CLEC12A Targets the PI3K-Akt Pathway

As our phosphoproteomic analysis showed that CLEC12A regulated phosphorylation of members of the Akt pathway [PI3K, PKD1, and p38 MAPK; (30, 31)], we examined CLEC12A regulation of the Akt pathway in MSU-stimulated human neutrophils. **Figure 6B** shows that the peak of MSU-induced phosphorylation of Akt occurred earlier (30 sec vs 120 sec) in cells in which CLEC12A was internalized by cross-linking with 50C1 and an anti-F(ab’)_2_ antibody. As previously reported (31), **Supplementary Figure 5** shows that the p38 MAPK inhibitor, SB203580, blocked MSU-induced phosphorylation of Akt. Thus, the effect of CLEC12A internalization on MSU-stimulated p38 MAPK activation was determined. **Figure 6C** shows that MSU stimulated a low level of p38 MAPK phosphorylation at 60 sec and 120 sec. Reduced CLEC12A expression significantly enhanced MSU-stimulated p38 MAPK phosphorylation at those same time points. A similar enhancement of MSU-induced p38 MAPK and Akt phosphorylation was observed after knocking-down CLEC12A expression in the monocytic cell line THP-1 (**Supplementary Figure 6**). CLEC12A thus negatively regulates PI3K/Akt pathway activation in MSU-stimulated neutrophils and potentially monocytes.

We previously reported that silencing CLEC12A in the neutrophil-like cell line (PLB-985) and the internalization of CLEC12A in human neutrophils enhances MSU-induced release of IL-8 (9). To determine if CLEC12A inhibition of the PI3K/p38 MAPK/Akt pathway alters MSU-induced neutrophil responses, we measured MSU-stimulated IL-8 production and release. Wortmannin and Ly294002 significantly inhibited the MSU-induced release of IL-8 by human neutrophils (**Figure 6D**) as does the p38 inhibitor SB203580 (**Supplementary Figure 7** and Tatsiy et al). CLEC12A thus inhibits IL-8 release in MSU-stimulated human neutrophils, in part, through regulation of the PI3K/Akt pathway.

## Discussion

The current study identifies key signal transduction events involved in CLEC12A regulation of neutrophil activity. Following clustering on the surface of human neutrophils, CLEC12A translocates to flotillin-rich membrane domains, where the ITIM domain undergoes phosphorylation by a Src-family kinase. CLEC12A clustering results in altered phosphorylation of a number of kinases and their substrates, primarily in MAPK signal transduction pathways. CLEC12A also regulates the ability of TNF-α and MSU to activate a number of kinase pathways, including those containing JNK, p38 MAPK, Src family non-receptor tyrosine kinases, and phosphoinositol kinases. We identified a novel role for CLEC12A in the regulation of the p38 MAPK-PI3K-Akt axis in human neutrophils, one of the pathways that regulates cytokine production stimulated by MSU.

One of the earliest events of inhibitory receptor signaling is the phosphorylation of the ITIM (9). This is the first report providing direct evidence that phosphorylation of the CLEC12A ITIM domain depends on receptor clustering in flotillin-rich membrane domains. We showed translocation of CLEC12A to those membrane domains using membrane fractionation, confocal microscopy, and biochemical disruption with methyl-β-cyclodextrin. The key role of those membrane domains as signaling hubs suggests a mechanism by which CLEC12A regulates the signaling of other neutrophil activating receptors that also translocate to those domains. Further studies to identify CLEC12A binding partners within flotillin-rich, membrane domains should clarify the range of CLEC12A regulation of neutrophil activation.

Internalization of CLEC12A is a major mechanism preventing the counter-regulatory activity of this inhibitory receptor in neutrophil (9). CLEC12A clustering in membrane domains is necessary for internalization and thus required for both the activation and inactivation of CLEC12A. We envision a sequence of events where ligand-induced receptor cross-linking leads to translocation to flotillin-rich membrane domains, where the CLEC12A ITIM domain is phosphorylated by Src tyrosine kinases within seconds. That phosphorylation recruits phosphatases localized within those membrane domains, limiting phosphorylation-dependent activation of signal transduction components localized within membrane domains that are required for neutrophil functional responses. This inhibitory process is terminated by internalization of the membrane domain complex 10-20 minutes later (9). Our data show that CLEC12A internalization is partly dependent on microtubules, consistent with previous reports on flotillin recycling (32). This may explain the efficacy of the microtubule inhibitor, colchicine, in the treatment of gout. Inhibition of CLEC12A internalization by colchicine would maintain CLEC12A plasma membrane expression and inhibition of cell activation.

Our unbiased phosphoproteomic analysis identified a change in the phosphorylation status of a number of protein kinases and phosphatases induced by CLEC12A cross-linking with molecular functions that included GTPase regulation, phospholipid binding, cell adhesion, and actin binding. Prominent among the kinases were upstream components of the ERK, JNK, and p38 MAPK pathways. The regulation of components of the MAPK pathway may be a recurring theme in CLEC12A biology as CLEC12A cross-linking induces the phosphorylation of p38 MAPK in bone marrow-derived dendritic cells (5). CLEC12A also negatively regulates the expression of MAPKAPK5 in bone marrow-derived macrophages of CLEC12A KO mice (33). Potentially important to the regulatory role of CLEC12A, receptor cross-linking resulted in phosphorylation of 4 phosphatases. CLEC12A cross-linking also induced a change in the phosphorylation of components of a number of signal transduction pathways activated by TNF-α in human neutrophils. TNF-α is a cytokine that regulates a number of pro-inflammatory neutrophil functions, including granule mobilization, respiratory burst activity, and cytokine synthesis, and also plays a key role in chronic inflammatory diseases such as rheumatoid arthritis and gout (34). The molecular functions of these pathways included cytoskeletal regulation, GTPase activity, and phospholipid binding. Kinase phosphorylation inhibited by CLEC12A included two JNKs (MAPK8, MAPK10), PAK1/2, and several cyclin-dependent kinases, which regulate neutrophil transcription, apoptosis, migration, and NET formation (35–37). Additional TNF-α signaling components inhibited by CLEC12A included kinases (PIK3CG, PDPK1, PAK1), and phosphatases (PTEN) that regulate the PI3K/Akt pathway. MSU-stimulated human neutrophils demonstrated similarities in signal transduction pathway component phosphorylation to that following antibody cross-linking of CLEC12A. We provide evidence that CLEC12A negatively regulates the MSU-induced serine phosphorylation of PKC substrates and the PI3K pathway. We also show that CLEC12A inhibits MSU-induced phosphorylation of Akt and p38 MAPK. As MSU-stimulated Akt phosphorylation is dependent on p38 MAPK activity, our results suggest that CLEC12A regulation of p38/PI3K/pAkt in MSU-activated human neutrophils is important for regulation of neutrophil functional responses. To link CLEC12A regulation of MSU-induced signaling to neutrophil functional responses, MSU-induced release of IL-8 by human neutrophils was examined. We showed previously that silencing of CLEC12A in a neutrophil-like cell line increased MSU-induced release of IL-8 (9). Moreover, our data show that MSU-induced synthesis and release of IL-8 is dependent on PI3K and p38. We thus identified one of the MSU-activated pathways, the p38 MAPK/PI3K/Akt pathway, that CLEC12A attenuates to inhibit MSU-induced IL-8 release. In contrast, CLEC12A does not modulate degranulation by human neutrophils in response to MSU *(our preliminary data)*, additional evidence for the selective regulation of a subset of MSU-induced signaling events and functions in neutrophils.

Inhibitory receptors regulate cell function by recruiting phosphatases that dephosphorylate activating signal transduction pathways (12–17). CLEC12A recruits the phosphatases SHP-1 and SHP-2 in transfected, pervanadate-treated RAW cells (8), and SHP-2 is recruited by CLEC12A in transfected HEK-293T cells treated with pervanadate *(data not shown)*. These phosphatases, however, were not identified in our phosphoproteomic screen, indicating they are either not involved in CLEC12A signaling in neutrophils or their interaction with CLEC12A could not be detected under our experimental conditions. The phosphoproteomic data did identify an alternative downstream candidate known to dampen cell activation by inhibiting Src-family kinases, CSK. Further studies are required to confirm phosphatase recruitment by CLEC12A in human neutrophils and to identify the phosphatase(s) involved in CLEC12A function.

**Figure 7:**
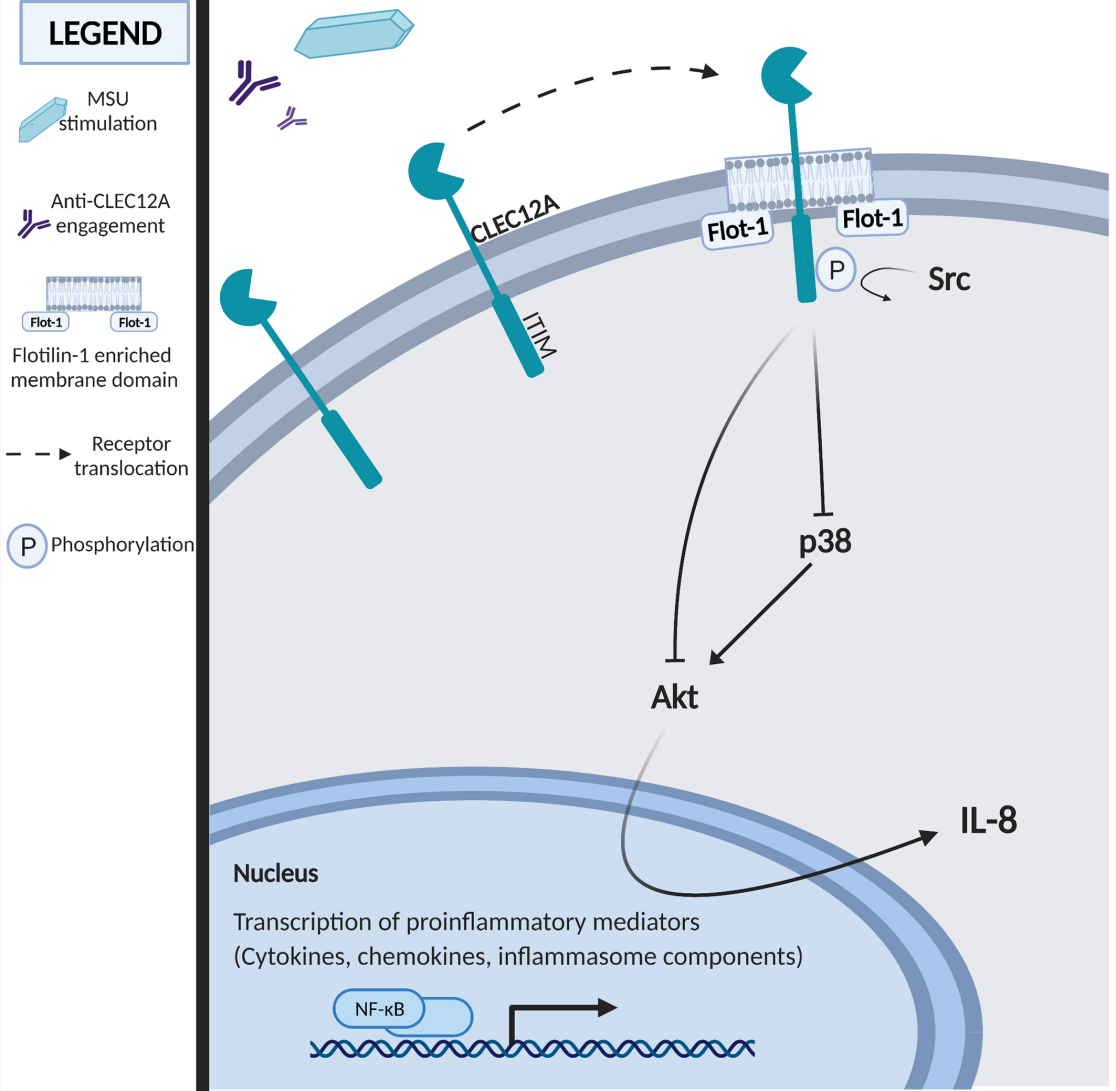
Schematic representation of the MSU-induced signaling pathways negatively regulated by CLEC12A upon its translocation to flotillin-rich membrane domains and its internalization.

Our observations suggest that CLEC12A plays a counter-regulatory role in the MSU stimulation of neutrophils through inhibition of signal transduction pathways containing p38 MAPK, PI3K, and Akt. Although our data show that both p38 MAPK and PI3K are upstream of Akt activation by MSU in human neutrophils, each of those signaling components contribute to multiple signal transduction pathways. While we show that CLEC12A regulation of the PI3K/Akt pathway controls IL-8 synthesis and release, the ability of these pathways to mediate CLEC12A regulation of other neutrophil functions remains to be established. PI3K, for instance, also regulates neutrophil recruitment, survival and activation (38, 39). **Figure 7** presents a preliminary model of CLEC12A regulation of neutrophil activation based on our data. Whilst we interpret our data on the regulation of MSU-induced signaling by CLEC12A as a linear series of events, it is likely that the regulatory pathways interact with a variety of other signaling proteins in a non-linear fashion. Confirmation of the regulation of phosphorylation events identified by our phosphoproteomic screen will require a comprehensive analysis of the molecular pathways and functional responses stimulated by MSU. The ability of CLEC12A to regulate signal transduction pathways activated by TNF-α suggests our findings are applicable to other inflammatory diseases, such as rheumatoid arthritis. Understanding the molecular mechanisms by which CLEC12A inhibits neutrophil functional responses may provide new therapeutic strategies to address chronic inflammatory diseases.

## Data Availability Statement

The raw data supporting the conclusions of this article will be made available by the authors, without undue reservation.

## Ethics Statement

The studies involving human participants were reviewed and approved by CHU de Québec-Université Laval research ethics committee (2012-337). The patients/participants provided their written informed consent to participate in this study.

## Author Contributions

Conceptualization: MF, KM, and PN. Methodology, investigation, and formal analysis: major contribution by GP, KM, and MM, and contribution by JV, MV, YS, AM and MF (formal analysis). Review and editing, and visualization: major contribution by KM and MF, and contribution by GP, MV, JV, SE, MHL, and PN. Funding acquisition, project administration, and writing original draft: MF. All authors contributed to the article and approved the submitted version.

## Funding

This work was supported by a Canadian Institutes of Health Research (CIHR) grant (number 142408) and funds from the CHU de Québec Foundation awarded to MF. MF also received an Arthritis Society Investigator Award. JV received a scholarship from the CHU de Québec Foundation and the ‘Fonds Pierre-Borgeat sur l’Arthrites et les Maladies Rhumatismales’.

## Conflict of Interest

The authors declare that the research was conducted in the absence of any commercial or financial relationships that could be construed as a potential conflict of interest.

## Abbreviations

ITAM, immunoreceptor tyrosine-based activation motif; ITIM, immunoreceptor tyrosine-based inhibition motif; PI3K, phosphatidylinositol 3’ kinase; PP2, 4-amino-5-(4-chlorophenyl)-7(t-butyl)pyrazolo(3,4-*d*]pyrimidine; PP3, 4-amino-1-phenylpyrazol(3,4-*d*)pyrimidine; MSU, monosodium urate crystals.
