# Supplementary material for: The Inhibitory Receptor CLEC12A Regulates PI3K-Akt Signaling to Inhibit Neutrophil Activation and Cytokine Release
Source: Front Immunol. 2021 Jun 21;12:650808. doi: 10.3389/fimmu.2021.650808 (PMC8256872; doi:10.3389/fimmu.2021.650808)
Supplement: Supplementary file 1 [file DataSheet_1.pdf]

Supplementary Material

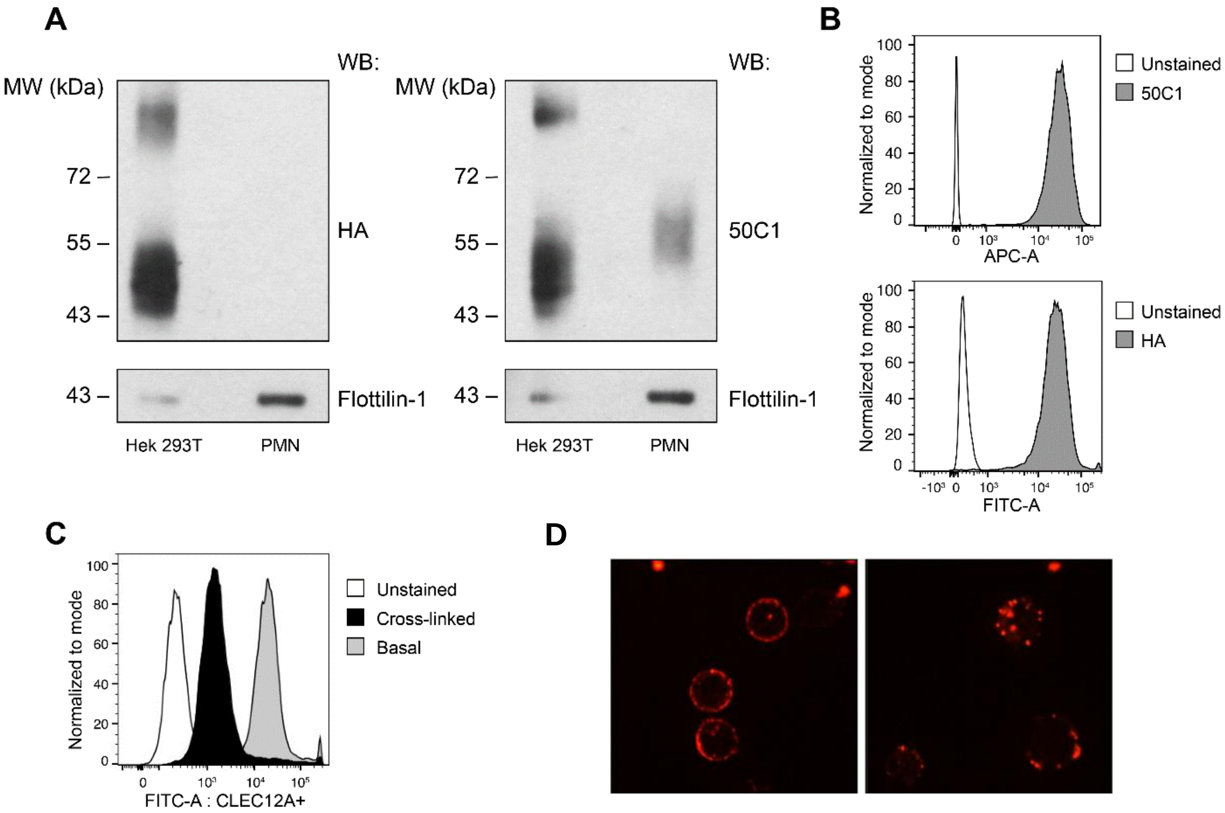
A
MW (kDa)
WB:
72 –
55 –
43 –
HA
50C1
43 –
Flottilin-1
Hek 293T
PMN
B
Normalized to mode
APC-A
FITC-A
Unstained
50C1
HA
C
Normalized to mode
Unstained
Cross-linked
Basal
FITC-A : CLEC12A+
D

E

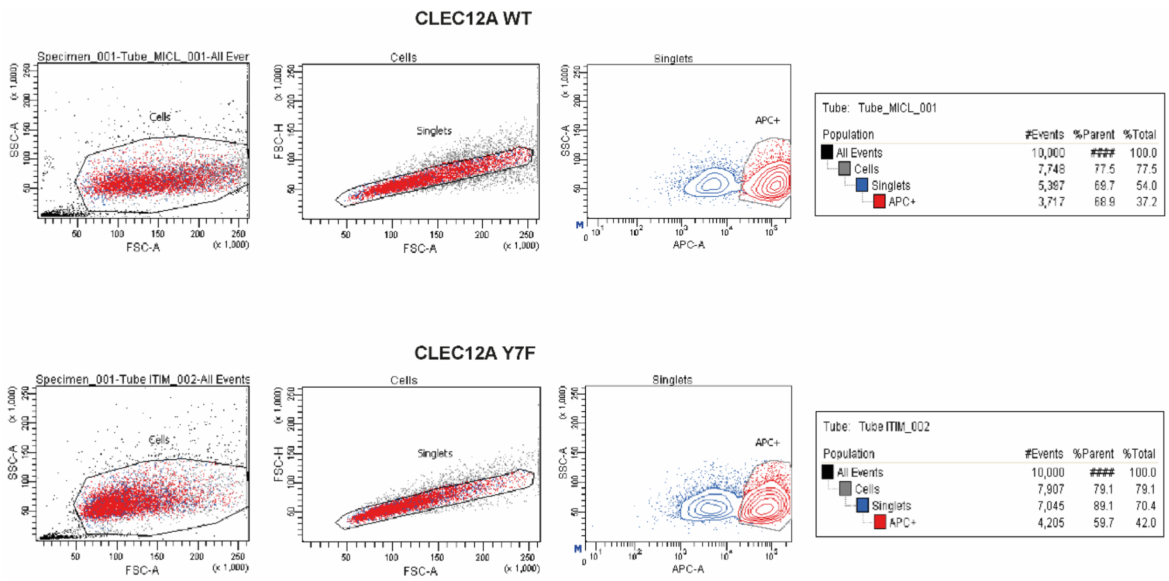
CLEC12A WT
Cells
Singlets
APC+
SSC-A
FSC-A
FSC-H
APC-A
Tube: Tube_MICL_001
Population #Events %Parent %Total
All Events 10,000 #### 100.0
Cells 7,748 77.5 77.5
Singlets 5,397 69.7 54.0
APC+ 3,717 68.9 37.2
CLEC12A Y7F
Tube: Tube ITIM_002
Population #Events %Parent %Total
All Events 10,000 #### 100.0
Cells 7,907 79.1 79.1
Singlets 7,045 89.1 70.4
APC+ 4,205 59.7 42.0

Supplementary Figure 1. HEK-293T cells express CLEC12A-HA-wt on their surface that can be internalised by antibody-induced cross-linking as in human neutrophils. *(A)* Total cell lysates were prepared from HEK-293T cells transiently transfected with CLEC12A-HA-wt or from human neutrophils isolated from healthy donors as described in *Materials and Methods* prior to immunoblotting with an anti-HA antibody or 50C1 antibodies, *left* and *right* gels, respectively. The anti-flotillin-1 antibody was used as a loading control. *(B)* Cell-surface expression of CLEC12Awt-HA was confirmed by flow cytometry with the same antibodies conjugated to the indicated fluorochromes. *(C)* The internalisation of CLEC12Awt-HA expressed on transiently transfected HEK-293T was determined by flow cytometry after cross-linking with an anti-HA and an anti-$F(ab')_2$ secondary antibody as described in *Materials and Methods*. These data are representative of 3 experiments. Similar observations were made in HeLa cells *(data not shown)*. *(D)* The internalisation of CLEC12A in human neutrophils induced by fluorescently-labelled 50C1 after incubation at 37^0C *(right image)* for 10 minutes. Cell-surface CLEC12A staining without internalisation is shown in the *left image*. Images were acquired at 63X with the Quorum WAVFX spinning disc system (Quorum Technologies, Guelph, Ontario, Canada) and images analyzed with the Volocity quantitation module as previously described in Gagné(9). *(E)* Gating strategy for the sorting of cells stably transfected with CLEC12A by flow cytometry using an APC-labeled CLEC12A antibody.

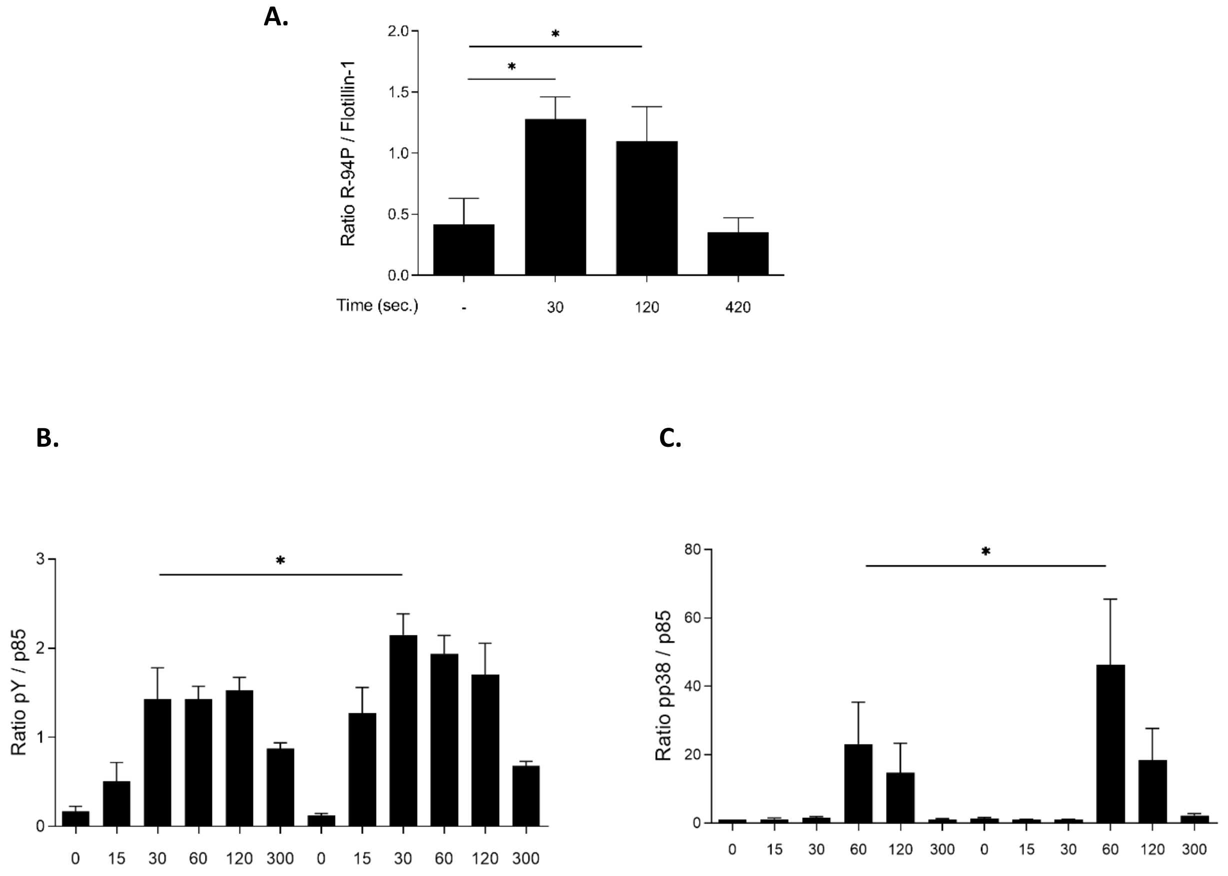

Supplementary Figure 2. Quantification of Western blots by densitometry**-** Densitometry analysis was performed with Image Lab software (Bio-Rad) on blots in Figure 4a *(A)* and Figure 6b *((B), anti-tyrosine phosphorylation bands >72 kd; (C), p38 blot)* of the main manuscript. Statistical analysis: Two way ANOVA and the Dunnett's mutliple comparisons test was performed to compare the treated cells to the control cells in *(A)* and the paired t-test was performed in *(B)* and *(C)*. * P <0.05.

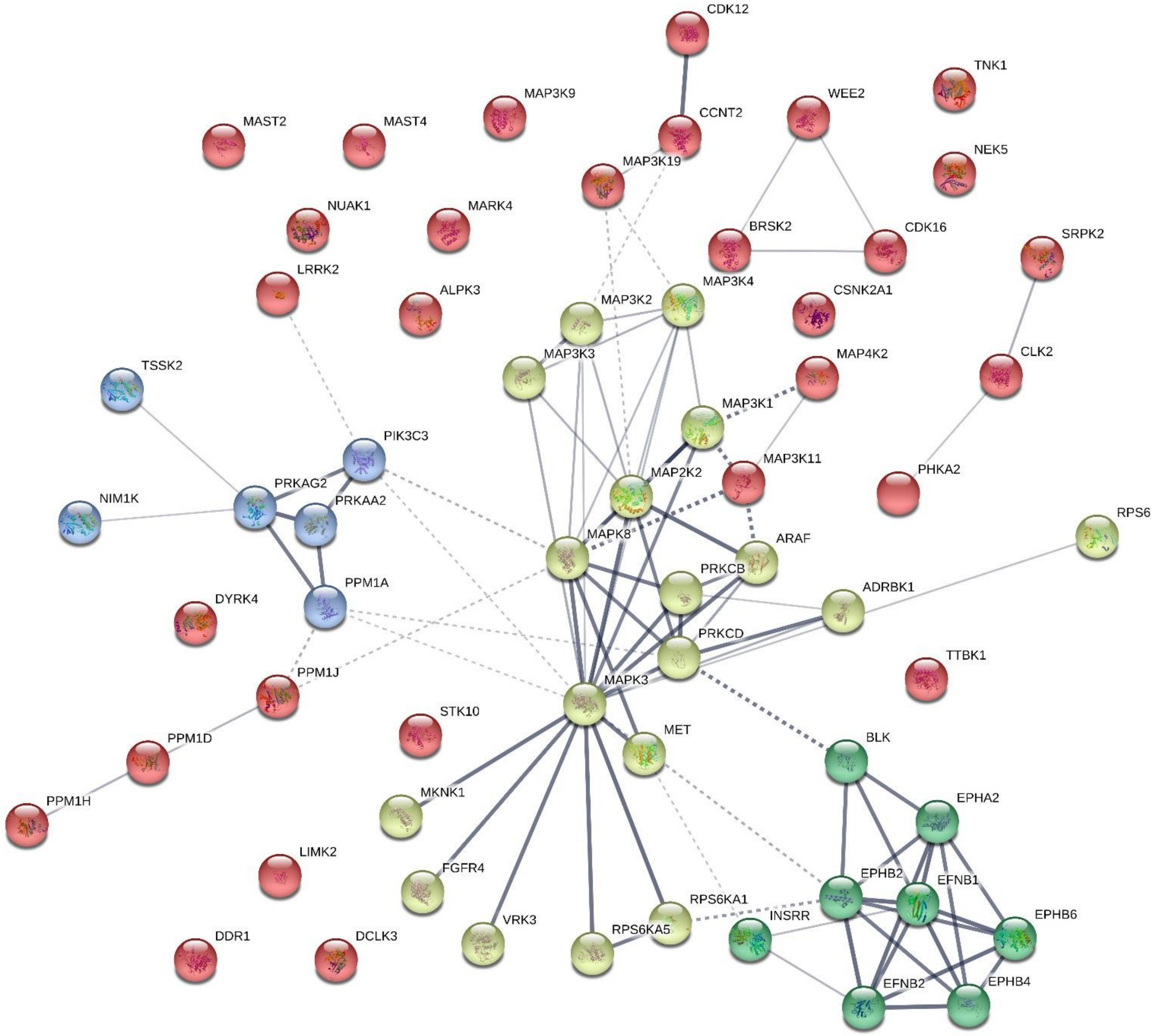

Supplementary Figure 3. STRING analysis of proteins with increased phosphorylation after CLEC12A cross-linking compared to isotype cross-linking.

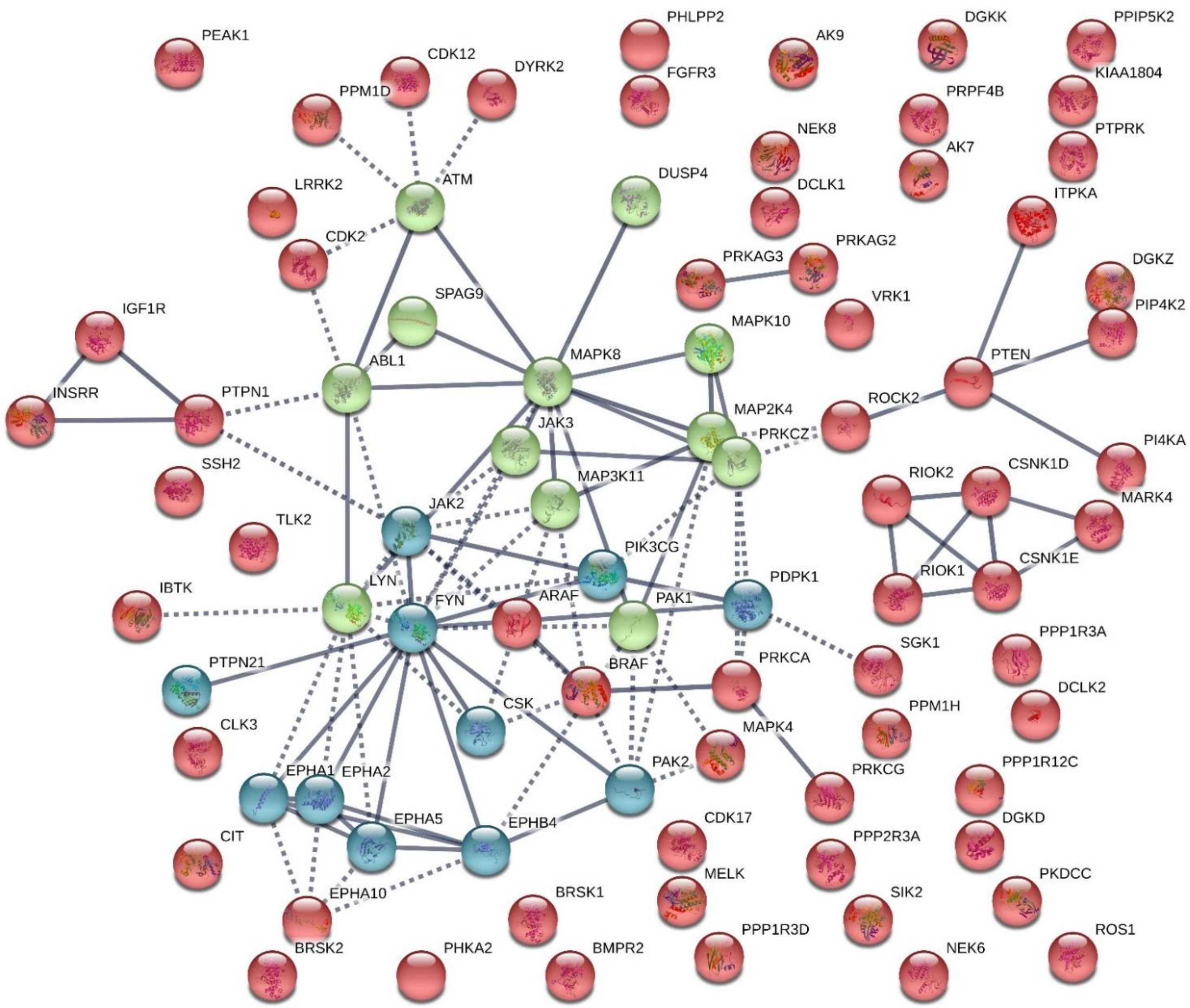

Supplementary Figure 4. STRING analysis of kinases with increased phosphorylation in human neutrophils stimulated with TNF-α compared to neutrophils stimulated with TNF- α and cross-linked for CLEC12A cross-linking.

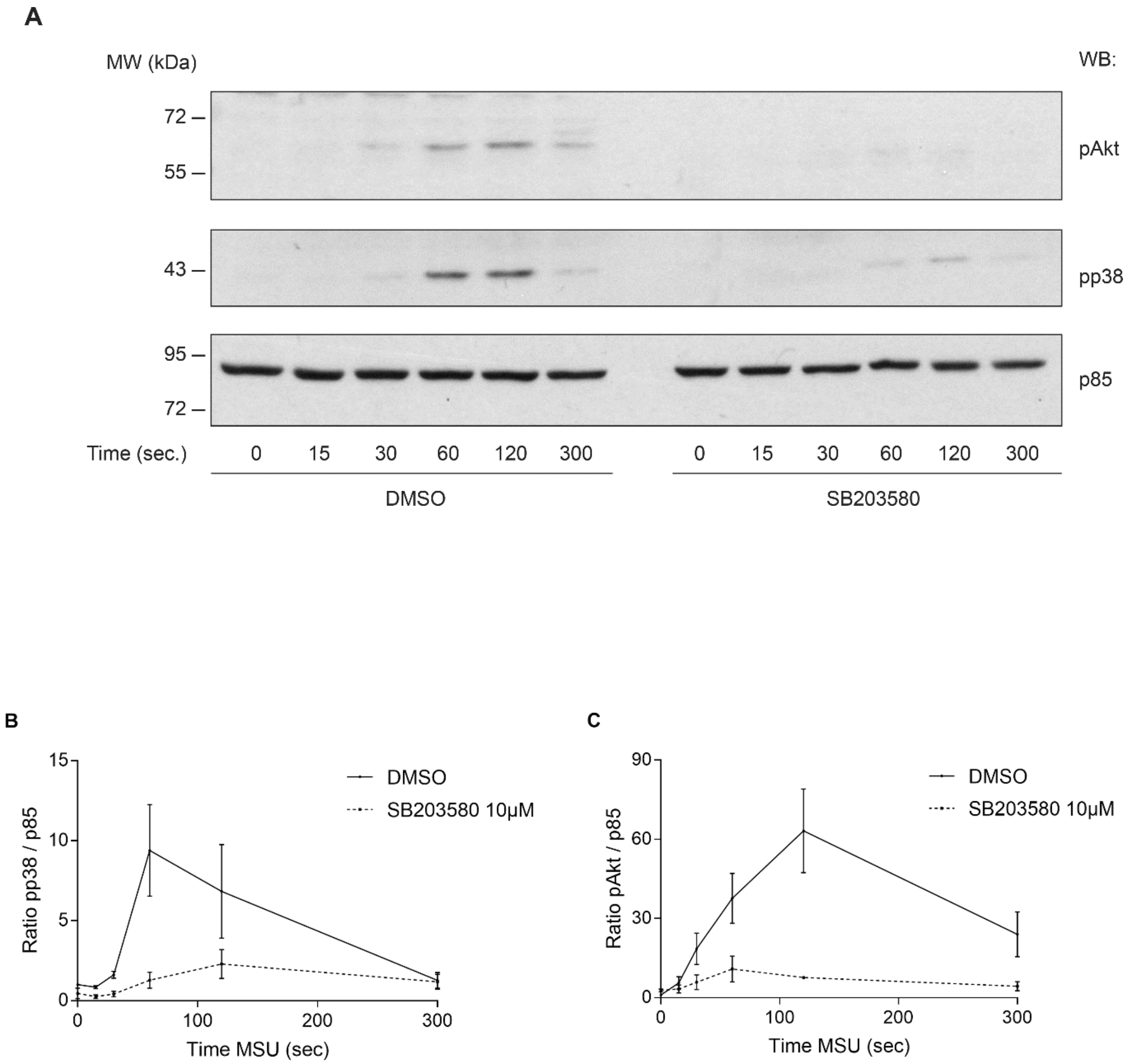

Supplementary Figure 5. MSU induce the phosphorylation of Akt via p38 in human neutrophils. *(A)* Neutrophils were stimulated with MSU in the presence or absence of SB203580 as described in *Materials and Methods*. Cell lysates were then immunoblotted with an anti-phospho Akt *(pAkt)* or anti-phospho-p38 *(pp38)* antibody as well as an anti-PI3K p85 subunit antibody as a loading control. *(B & C)* Densitometry analysis of the immunoblots in *(A)*. These data are representative of 3 independent experiments.

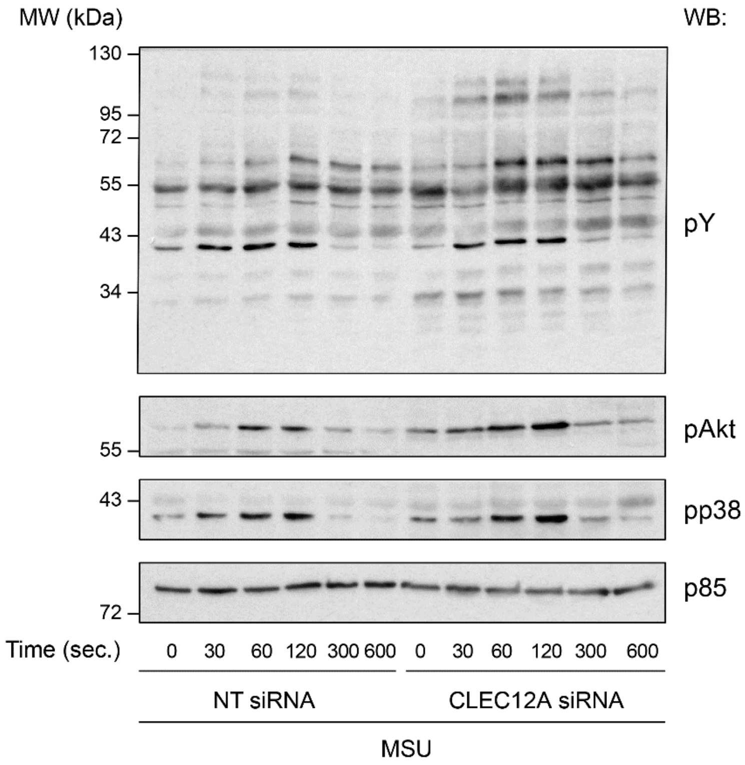

Supplementary Figure 6. CLEC12A knock-down in THP-1 cells enhances MSU-induced global tyrosine phosphorylation and the phosphorylation of Akt and p38. The monocytic cell line, THP-1, was cultured in RPMI supplemented with 10% heat-inactivated fetal calf or bovine serum. THP-1 cells were transfected with CLEC12A siRNA prior to stimulation with MSU as described in *Materials and Methods* followed by immunoblotting with an anti-phoshotyrosine (pY), anti-phospho Akt and a PI3K p85 subunit (loading control) antibody. These data are representative of 3 experiments.

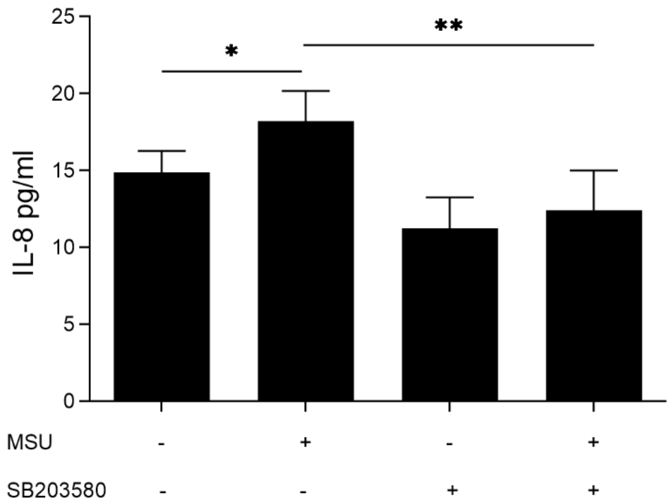

Supplementary Figure 7. MSU-induced IL-8 production in human neutrophils is p38-dependent. Human neutrophils were stimulated with 0.3 mg/ml MSU in the presence or absence of SB203580 or diluant (*(-)*; DMSO) and released CXCL8/IL-8 determined by ELISA. Data are representative of 3 independent experiments. Statistical analysis: Two way ANOVA and the Turkey's mutliple comparisons test was performed to compare the treated cells to the control cells. * P <0.05, ** P < 0.01.

Table S1. GO molecular functions of CLEC12A-Induced Phosphoproteins

Enrichment of phosphoproteins CLEC12A>isotype GO molecular function

	Homo sapiens (REF)	CLEC12A-induced					
GO molecular function	#	#	Expected	Fold Enrichment	+/-	Raw P value	FDR
MAP kinase kinase kinase activity	28	9	1.74	5.18	+	2.18E-04	2.90E-02
protein kinase activity	614	64	38.13	1.68	+	1.65E-04	2.40E-02
guanyl-nucleotide exchange factor activity	218	30	13.54	2.22	+	1.72E-04	2.43E-02
GTPase binding	545	62	33.85	1.83	+	2.10E-05	3.75E-03
GTPase regulator activity	303	37	18.82	1.97	+	2.63E-04	3.30E-02
phospholipid binding	426	49	26.46	1.85	+	1.45E-04	2.26E-02
cell adhesion molecule binding	483	55	30	1.83	+	5.46E-05	9.42E-03
actin binding	435	49	27.02	1.81	+	1.76E-04	2.41E-02
calcium ion binding	715	73	44.41	1.64	+	1.01E-04	1.62E-02

Table S2. Protein Kinases and Phosphatases Phosphorylated upon CLEC12A Cross-linking			
Protein	Gene	Protein	Gene
Beta-adrenergic receptor kinase 1	ADRBK1	Mitogen-activated protein kinase kinase kinase kinase 2	MAP4K2
Alpha-protein kinase 3	ALPK3	Mitogen-activated protein kinase 3	MAPK3
Serine/threonine-protein kinase A-Raf	ARAF	Mitogen-activated protein kinase 8	MAPK8
Tyrosine-protein kinase Blk	BLK	MAP/microtubule affinity-regulating kinase 4	MARK4
Serine/threonine-protein kinase BRSK2	BRSK2	Microtubule-associated serine/threonine-protein kinase 2	MAST2
Cyclin-T2	CCNT2	Microtubule-associated serine/threonine-protein kinase 4	MAST4
Cyclin-dependent kinase 12	CDK12	Hepatocyte growth factor receptor	MET
Cyclin-dependent kinase 16	CDK16	MAP kinase-interacting serine/threonine-protein kinase 1	MKNK1
Dual-specificity protein kinase CLK2	CLK2	Serine/threonine-protein kinase Nek5	NEK5
Casein kinase II subunit alpha	CSNK2A1	Serine/threonine-protein kinase NIM1	NIM1K
Serine/threonine-protein kinase DCLK3	DCLK3	AMPK-related protein kinase 5	NUAK1
Epithelial discoidin domain-containing receptor 1	DDR1	Phosphorylase b kinase regulatory subunit alpha	PHKA2
Dual-specificity tyrosine-phosphorylation-regulated kinase 4	DYRK4	Phosphatidylinositol 3-kinase catalytic subunit type 3	PIK3C3
Ephrin-B1	EFNB1	Protein phosphatase 1A	PPM1A

Ephrin-B2	EFNB2	Protein phosphatase 1D	PPM1D
Ephrin type-A receptor 2	EPHA2	Protein phosphatase 1H	PPM1H
Ephrin type-B receptor 2	EPHB2	Protein phosphatase 1J	PPM1J
Ephrin type-B receptor 4	EPHB4	5'-AMP-activated protein kinase subunit alpha-2	PRKAA2
Ephrin type-B receptor 6	EPHB6	5'-AMP-activated protein kinase subunit gamma-2	PRKAG2
Fibroblast growth factor receptor 4	FGFR4	Protein kinase C beta	PRKCB
Insulin receptor-related protein	INSRR	Protein kinase C delta	PRKCD
LIM domain kinase 2	LIMK2	Ribosomal protein S6 kinase alpha-1	RPS6KA1
Leucine-rich repeat serine/threonine-protein kinase 2	LRRK2	Ribosomal protein S6 kinase alpha-5	RPS6KA5
Mitogen-activated protein kinase kinase 2	MAP2K2	Ribosomal protein S6 kinase delta-1	RPS6KC1
Mitogen-activated protein kinase kinase kinase 1	MAP3K1	SRSF protein kinase 2	SRPK2
Mitogen-activated protein kinase kinase kinase 11	MAP3K11	Serine/threonine-protein kinase 10	STK10
Mitogen-activated protein kinase kinase kinase 19	MAP3K19	Non-receptor tyrosine kinase TNK1	TNK1
Mitogen-activated protein kinase kinase kinase 2	MAP3K2	Testis-specific serine/threonine-protein kinase 2	TSSK2
Mitogen-activated protein kinase kinase kinase 3	MAP3K3	Tau-tubulin kinase 1	TTBK1

Mitogen-activated protein kinase kinase kinase 4	MAP3K4	Inactive serine/threonine-protein kinase VRK3	VRK3
Mitogen-activated protein kinase kinase kinase 9	MAP3K9	Wee1-like protein kinase 2	WEE2

Table S3. Molecular Functions of TNF-α Phosphoproteins Reduced by CLEC12A Cross-linking

	Homo sapiens	Current List				
GO molecular function	#	#	Expected	Fold Enrichment	P value	FDR
microtubule motor activity	86	18	5.27	3.41	2.64E-05	2.02E-03
tau-protein kinase activity	22	8	1.35	5.93	2.26E-04	1.42E-02
protein kinase activity	614	71	37.64	1.89	1.68E-06	1.73E-04
dynein complex binding	23	8	1.41	5.67	2.89E-04	1.66E-02
dynein intermediate chain binding	29	9	1.78	5.06	2.47E-04	1.53E-02
ionotropic glutamate receptor binding	34	10	2.08	4.8	1.63E-04	1.07E-02
glutamate receptor binding	50	13	3.06	4.24	5.24E-05	3.69E-03
alpha-actinin binding	32	9	1.96	4.59	4.54E-04	2.55E-02
histone-lysine N-methyltransferase activity	44	10	2.7	3.71	9.30E-04	4.65E-02
structural constituent of cytoskeleton	99	21	6.07	3.46	4.85E-06	4.51E-04
Rac GTPase binding	69	14	4.23	3.31	2.67E-04	1.60E-02
Rho GTPase binding	180	29	11.03	2.63	1.45E-05	1.19E-03
Ras GTPase binding	431	56	26.42	2.12	9.72E-07	1.05E-04
methylated histone binding	66	13	4.05	3.21	5.60E-04	3.03E-02

Rho guanyl-nucleotide exchange factor activity	82	16	5.03	3.18	1.49E-04	9.91E-03
Ras guanyl-nucleotide exchange factor activity	140	23	8.58	2.68	6.52E-05	4.53E-03
microtubule binding	241	47	14.77	3.18	1.03E-10	2.00E-08
actin filament binding	194	34	11.89	2.86	3.60E-07	4.41E-05
thiol-dependent ubiquitin-specific protease activity	107	17	6.56	2.59	7.82E-04	4.05E-02
calmodulin binding	198	31	12.14	2.55	1.02E-05	8.76E-04
ion channel binding	128	20	7.85	2.55	4.87E-04	2.70E-02
protein tyrosine kinase activity	136	20	8.34	2.4	7.70E-04	4.03E-02
cadherin binding	315	44	19.31	2.28	3.02E-06	2.99E-04
protein kinase binding	658	78	40.33	1.93	2.01E-07	2.59E-05
protein serine/threonine kinase activity	446	52	27.34	1.9	3.69E-05	2.73E-03
GTPase regulator activity	303	35	18.57	1.88	8.68E-04	4.44E-02
phospholipid binding	426	48	26.11	1.84	1.96E-04	1.25E-02
RNA binding	1661	152	101.81	1.49	2.67E-06	2.70E-04

**Table S4. Protein Kinases and Phosphatases with Reduced Phosphorylation following CLEC12A Cross-linking**			
Protein	Gene	Protein	Gene
Abelson murine leukemia viral oncogene homolog 1	ABL1	maternal embryonic leucine zipper kinase	MELK
Adenylate kinase 7	AK7	Mitogen-activated protein kinase kinase kinase MLK4	MLK4
Adenylate kinase 9	AK9	Serine/threonine-protein kinase Nek6	NEK6
Serine/threonine-protein kinase A-Raf	ARAF	Serine/threonine-protein kinase Nek8	NEK8
Serine-protein kinase ATM	ATM	Serine/threonine-protein kinase PAK 1	PAK1
Bone morphogenetic protein receptor type-2	BMPR2	Serine/threonine-protein kinase PAK 2	PAK2
Serine/threonine-protein kinase B-raf	BRAF	Putative 3-phosphoinositide-dependent protein kinase 1	PDPK1
Serine/threonine-protein kinase BRSK1	BRSK1	Pseudopodium-enriched atypical kinase 1	PEAK1
Serine/threonine-protein kinase BRSK2	BRSK2	Phosphorylase b kinase regulatory subunit alpha	PHKA2
Cyclin-dependent kinase 12	CDK12	PH domain leucine-rich repeat-containing protein phosphatase 2	PHLPP2
Cyclin-dependent kinase 17	CDK17	Phosphatidylinositol 4-kinase alpha	PI4KA
Cyclin-dependent kinase 2	CDK2	Phosphatidylinositol 4,5-bisphosphate 3-kinase catalytic subunit gamma	PIK3CG

citron rho-interacting serine/threonine kinase	CIT	Phosphatidylinositol 5-phosphate 4-kinase type-2 alpha	PIP4K2A
CDC-like kinase 3	CLK3	Protein kinase domain-containing protein, cytoplasmic	PKDCC
Tyrosine-protein kinase CSK	CSK	Inositol hexakisphosphate and diphosphoinositol-pentakisphosphate kinase 2	PPIP5K2
Casein kinase I isoform delta	CSNK1D	Protein phosphatase 1D	PPM1D
Casein kinase I isoform epsilon	CSNK1E	Protein phosphatase 1H	PPM1H
Doublecortin and CaM kinase-like 1	DCLK1	Protein phosphatase 1 regulatory subunit 12C	PPP1R12 C
Doublecortin and CaM kinase-like 2	DCLK2	Protein phosphatase 1 regulatory subunit 3A	PPP1R3A
Diacylglycerol kinase delta	DGKD	Protein phosphatase 1 regulatory subunit 3D	PPP1R3D
Diacylglycerol kinase kappa	DGKK	Phosphatase 2A regulatory subunit B subunit alpha	PPP2R3A
Diacylglycerol kinase zeta	DGKZ	5'-AMP-activated protein kinase subunit gamma-2	PRKAG2
Dual specificity protein phosphatase 4	DUSP4	5'-AMP-activated protein kinase subunit gamma-2	PRKAG2
Dual specificity tyrosine-phosphorylation-regulated kinase 2	DYRK2	5'-AMP-activated protein kinase subunit gamma-3	PRKAG3
Ephrin type-A receptor 1	EPHA1	5'-AMP-activated protein kinase subunit gamma-3	PRKAG3
Ephrin type-A receptor 1	EPHA1	Protein kinase C alpha type	PRKCA

Ephrin type-A receptor 10	EPHA10	Protein kinase C gamma type	PRKCG
Ephrin type-A receptor 2	EPHA2	Protein kinase C gamma type	PRKCG
Ephrin type-A receptor 5	EPHA5	Protein kinase C zeta type	PRKCZ
Ephrin type-B receptor 4	EPHB4	Putative serine/threonine-protein kinase PRKY	PRKY
Fibroblast growth factor receptor	FGFR3	Serine/threonine-protein kinase PRP4	PRPF4B
Tyrosine-protein kinase Fyn	FYN	Phosphatidylinositol 3,4,5-trisphosphate 3-phosphatase and dual-specificity protein phosphatase PTEN	PTEN
Inhibitor of Bruton tyrosine kinase	IBTK	Tyrosine-protein phosphatase non-receptor type	PTPN1
Tyrosine-protein kinase receptor	IGF1R	Tyrosine-protein phosphatase non-receptor type 21	PTPN21
Insulin receptor-related protein	INSRR	Receptor-type tyrosine-protein phosphatase kappa	PTPRK
Inositol-trisphosphate 3-kinase A	ITPKA	Serine/threonine-protein kinase RIO1	RIOK1
Tyrosine-protein kinase	JAK2	Serine/threonine-protein kinase RIO2	RIOK2
Tyrosine-protein kinase JAK3	JAK3	Rho-associated protein kinase 2	ROCK2
Leucine-rich repeat serine/threonine-protein kinase 2	LRRK2	Tyrosine-protein kinase receptor	ROS1
LYN tyrosine kinase	LYN	Serine/threonine-protein kinase Sgk1	SGK1
Dual-specificity mitogen-activated protein kinase kinase 4	MAP2K 4	Serine/threonine-protein kinase SIK2	SIK2

Mitogen-activated protein kinase kinase kinase 11	MAP3K 11	C-Jun-amino-terminal kinase-interacting protein 4	SPAG9
mitogen-activated protein kinase 10	MAPK1 0	Protein phosphatase Slingshot homolog 2	SSH2
Mitogen-activated protein kinase 4	MAPK4	Serine/threonine-protein kinase tousled-like 2	TLK2
Mitogen-activated protein kinase 8	MAPK8	Serine/threonine-protein kinase VRK1	VRK1
MAP/microtubule affinity-regulating kinase 4	MARK4		
